# Search for Higgs boson pair production in the $$\gamma \gamma WW^*$$ channel using $$pp$$ collision data recorded at $$\sqrt{s}$$$$=13$$ TeV with the ATLAS detector

**DOI:** 10.1140/epjc/s10052-018-6457-x

**Published:** 2018-12-11

**Authors:** M. Aaboud, G. Aad, B. Abbott, O. Abdinov, B. Abeloos, D. K. Abhayasinghe, S. H. Abidi, O. S. AbouZeid, N. L. Abraham, H. Abramowicz, H. Abreu, Y. Abulaiti, B. S. Acharya, S. Adachi, L. Adamczyk, J. Adelman, M. Adersberger, A. Adiguzel, T. Adye, A. A. Affolder, Y. Afik, C. Agheorghiesei, J. A. Aguilar-Saavedra, F. Ahmadov, G. Aielli, S. Akatsuka, T. P. A. Åkesson, E. Akilli, A. V. Akimov, G. L. Alberghi, J. Albert, P. Albicocco, M. J. Alconada Verzini, S. Alderweireldt, M. Aleksa, I. N. Aleksandrov, C. Alexa, G. Alexander, T. Alexopoulos, M. Alhroob, B. Ali, G. Alimonti, J. Alison, S. P. Alkire, C. Allaire, B. M. M. Allbrooke, B. W. Allen, P. P. Allport, A. Aloisio, A. Alonso, F. Alonso, C. Alpigiani, A. A. Alshehri, M. I. Alstaty, B. Alvarez Gonzalez, D. Álvarez Piqueras, M. G. Alviggi, B. T. Amadio, Y. Amaral Coutinho, L. Ambroz, C. Amelung, D. Amidei, S. P. Amor Dos Santos, S. Amoroso, C. S. Amrouche, C. Anastopoulos, L. S. Ancu, N. Andari, T. Andeen, C. F. Anders, J. K. Anders, K. J. Anderson, A. Andreazza, V. Andrei, C. R. Anelli, S. Angelidakis, I. Angelozzi, A. Angerami, A. V. Anisenkov, A. Annovi, C. Antel, M. T. Anthony, M. Antonelli, D. J. A. Antrim, F. Anulli, M. Aoki, L. Aperio Bella, G. Arabidze, Y. Arai, J. P. Araque, V. Araujo Ferraz, R. Araujo Pereira, A. T. H. Arce, R. E. Ardell, F. A. Arduh, J-F. Arguin, S. Argyropoulos, A. J. Armbruster, L. J. Armitage, A. Armstrong, O. Arnaez, H. Arnold, M. Arratia, O. Arslan, A. Artamonov, G. Artoni, S. Artz, S. Asai, N. Asbah, A. Ashkenazi, E. M. Asimakopoulou, L. Asquith, K. Assamagan, R. Astalos, R. J. Atkin, M. Atkinson, N. B. Atlay, K. Augsten, G. Avolio, R. Avramidou, B. Axen, M. K. Ayoub, G. Azuelos, A. E. Baas, M. J. Baca, H. Bachacou, K. Bachas, M. Backes, P. Bagnaia, M. Bahmani, H. Bahrasemani, A. J. Bailey, J. T. Baines, M. Bajic, C. Bakalis, O. K. Baker, P. J. Bakker, D. Bakshi Gupta, E. M. Baldin, P. Balek, F. Balli, W. K. Balunas, J. Balz, E. Banas, A. Bandyopadhyay, S. Banerjee, A. A. E. Bannoura, L. Barak, W. M. Barbe, E. L. Barberio, D. Barberis, M. Barbero, T. Barillari, M-S. Barisits, J. Barkeloo, T. Barklow, N. Barlow, R. Barnea, S. L. Barnes, B. M. Barnett, R. M. Barnett, Z. Barnovska-Blenessy, A. Baroncelli, G. Barone, A. J. Barr, L. Barranco Navarro, F. Barreiro, J. Barreiro Guimarães da Costa, R. Bartoldus, A. E. Barton, P. Bartos, A. Basalaev, A. Bassalat, R. L. Bates, S. J. Batista, S. Batlamous, J. R. Batley, M. Battaglia, M. Bauce, F. Bauer, K. T. Bauer, H. S. Bawa, J. B. Beacham, M. D. Beattie, T. Beau, P. H. Beauchemin, P. Bechtle, H. C. Beck, H. P. Beck, K. Becker, M. Becker, C. Becot, A. Beddall, A. J. Beddall, V. A. Bednyakov, M. Bedognetti, C. P. Bee, T. A. Beermann, M. Begalli, M. Begel, A. Behera, J. K. Behr, A. S. Bell, G. Bella, L. Bellagamba, A. Bellerive, M. Bellomo, P. Bellos, K. Belotskiy, N. L. Belyaev, O. Benary, D. Benchekroun, M. Bender, N. Benekos, Y. Benhammou, E. Benhar Noccioli, J. Benitez, D. P. Benjamin, M. Benoit, J. R. Bensinger, S. Bentvelsen, L. Beresford, M. Beretta, D. Berge, E. Bergeaas Kuutmann, N. Berger, L. J. Bergsten, J. Beringer, S. Berlendis, N. R. Bernard, G. Bernardi, C. Bernius, F. U. Bernlochner, T. Berry, P. Berta, C. Bertella, G. Bertoli, I. A. Bertram, G. J. Besjes, O. Bessidskaia Bylund, M. Bessner, N. Besson, A. Bethani, S. Bethke, A. Betti, A. J. Bevan, J. Beyer, R. M. Bianchi, O. Biebel, D. Biedermann, R. Bielski, K. Bierwagen, N. V. Biesuz, M. Biglietti, T. R. V. Billoud, M. Bindi, A. Bingul, C. Bini, S. Biondi, T. Bisanz, J. P. Biswal, C. Bittrich, D. M. Bjergaard, J. E. Black, K. M. Black, R. E. Blair, T. Blazek, I. Bloch, C. Blocker, A. Blue, U. Blumenschein, Dr. Blunier, G. J. Bobbink, V. S. Bobrovnikov, S. S. Bocchetta, A. Bocci, D. Boerner, D. Bogavac, A. G. Bogdanchikov, C. Bohm, V. Boisvert, P. Bokan, T. Bold, A. S. Boldyrev, A. E. Bolz, M. Bomben, M. Bona, J. S. Bonilla, M. Boonekamp, A. Borisov, G. Borissov, J. Bortfeldt, D. Bortoletto, V. Bortolotto, D. Boscherini, M. Bosman, J. D. Bossio Sola, K. Bouaouda, J. Boudreau, E. V. Bouhova-Thacker, D. Boumediene, C. Bourdarios, S. K. Boutle, A. Boveia, J. Boyd, I. R. Boyko, A. J. Bozson, J. Bracinik, N. Brahimi, A. Brandt, G. Brandt, O. Brandt, F. Braren, U. Bratzler, B. Brau, J. E. Brau, W. D. Breaden Madden, K. Brendlinger, A. J. Brennan, L. Brenner, R. Brenner, S. Bressler, B. Brickwedde, D. L. Briglin, D. Britton, D. Britzger, I. Brock, R. Brock, G. Brooijmans, T. Brooks, W. K. Brooks, E. Brost, J. H Broughton, P. A. Bruckman de Renstrom, D. Bruncko, A. Bruni, G. Bruni, L. S. Bruni, S. Bruno, B. H. Brunt, M. Bruschi, N. Bruscino, P. Bryant, L. Bryngemark, T. Buanes, Q. Buat, P. Buchholz, A. G. Buckley, I. A. Budagov, M. K. Bugge, F. Bührer, O. Bulekov, D. Bullock, T. J. Burch, S. Burdin, C. D. Burgard, A. M. Burger, B. Burghgrave, K. Burka, S. Burke, I. Burmeister, J. T. P. Burr, D. Büscher, V. Büscher, E. Buschmann, P. Bussey, J. M. Butler, C. M. Buttar, J. M. Butterworth, P. Butti, W. Buttinger, A. Buzatu, A. R. Buzykaev, G. Cabras, S. Cabrera Urbán, D. Caforio, H. Cai, V. M. M. Cairo, O. Cakir, N. Calace, P. Calafiura, A. Calandri, G. Calderini, P. Calfayan, G. Callea, L. P. Caloba, S. Calvente Lopez, D. Calvet, S. Calvet, T. P. Calvet, M. Calvetti, R. Camacho Toro, S. Camarda, P. Camarri, D. Cameron, R. Caminal Armadans, C. Camincher, S. Campana, M. Campanelli, A. Camplani, A. Campoverde, V. Canale, M. Cano Bret, J. Cantero, T. Cao, Y. Cao, M. D. M. Capeans Garrido, I. Caprini, M. Caprini, M. Capua, R. M. Carbone, R. Cardarelli, F. C. Cardillo, I. Carli, T. Carli, G. Carlino, B. T. Carlson, L. Carminati, R. M. D. Carney, S. Caron, E. Carquin, S. Carrá, G. D. Carrillo-Montoya, D. Casadei, M. P. Casado, A. F. Casha, M. Casolino, D. W. Casper, R. Castelijn, F. L. Castillo, V. Castillo Gimenez, N. F. Castro, A. Catinaccio, J. R. Catmore, A. Cattai, J. Caudron, V. Cavaliere, E. Cavallaro, D. Cavalli, M. Cavalli-Sforza, V. Cavasinni, E. Celebi, F. Ceradini, L. Cerda Alberich, A. S. Cerqueira, A. Cerri, L. Cerrito, F. Cerutti, A. Cervelli, S. A. Cetin, A. Chafaq, D. Chakraborty, S. K. Chan, W. S. Chan, Y. L. Chan, P. Chang, J. D. Chapman, D. G. Charlton, C. C. Chau, C. A. Chavez Barajas, S. Che, A. Chegwidden, S. Chekanov, S. V. Chekulaev, G. A. Chelkov, M. A. Chelstowska, C. Chen, C. H. Chen, H. Chen, J. Chen, J. Chen, S. Chen, S. J. Chen, X. Chen, Y. Chen, Y-H. Chen, H. C. Cheng, H. J. Cheng, A. Cheplakov, E. Cheremushkina, R. Cherkaoui El Moursli, E. Cheu, K. Cheung, L. Chevalier, V. Chiarella, G. Chiarelli, G. Chiodini, A. S. Chisholm, A. Chitan, I. Chiu, Y. H. Chiu, M. V. Chizhov, K. Choi, A. R. Chomont, S. Chouridou, Y. S. Chow, V. Christodoulou, M. C. Chu, J. Chudoba, A. J. Chuinard, J. J. Chwastowski, L. Chytka, D. Cinca, V. Cindro, I. A. Cioară, A. Ciocio, F. Cirotto, Z. H. Citron, M. Citterio, A. Clark, M. R. Clark, P. J. Clark, C. Clement, Y. Coadou, M. Cobal, A. Coccaro, J. Cochran, A. E. C. Coimbra, L. Colasurdo, B. Cole, A. P. Colijn, J. Collot, P. Conde Muiño, E. Coniavitis, S. H. Connell, I. A. Connelly, S. Constantinescu, F. Conventi, A. M. Cooper-Sarkar, F. Cormier, K. J. R. Cormier, M. Corradi, E. E. Corrigan, F. Corriveau, A. Cortes-Gonzalez, M. J. Costa, D. Costanzo, G. Cottin, G. Cowan, B. E. Cox, J. Crane, K. Cranmer, S. J. Crawley, R. A. Creager, G. Cree, S. Crépé-Renaudin, F. Crescioli, M. Cristinziani, V. Croft, G. Crosetti, A. Cueto, T. Cuhadar Donszelmann, A. R. Cukierman, M. Curatolo, J. Cúth, S. Czekierda, P. Czodrowski, M. J. Da Cunha Sargedas De Sousa, C. Da Via, W. Dabrowski, T. Dado, S. Dahbi, T. Dai, F. Dallaire, C. Dallapiccola, M. Dam, G. D’amen, J. Damp, J. R. Dandoy, M. F. Daneri, N. P. Dang, N. D. Dann, M. Danninger, V. Dao, G. Darbo, S. Darmora, O. Dartsi, A. Dattagupta, T. Daubney, S. D’Auria, W. Davey, C. David, T. Davidek, D. R. Davis, E. Dawe, I. Dawson, K. De, R. De Asmundis, A. De Benedetti, S. De Castro, S. De Cecco, N. De Groot, P. de Jong, H. De la Torre, F. De Lorenzi, A. De Maria, D. De Pedis, A. De Salvo, U. De Sanctis, A. De Santo, K. De Vasconcelos Corga, J. B. De Vivie De Regie, C. Debenedetti, D. V. Dedovich, N. Dehghanian, M. Del Gaudio, J. Del Peso, D. Delgove, F. Deliot, C. M. Delitzsch, M. Della Pietra, D. Della Volpe, A. Dell’Acqua, L. Dell’Asta, M. Delmastro, C. Delporte, P. A. Delsart, D. A. DeMarco, S. Demers, M. Demichev, S. P. Denisov, D. Denysiuk, L. D’Eramo, D. Derendarz, J. E. Derkaoui, F. Derue, P. Dervan, K. Desch, C. Deterre, K. Dette, M. R. Devesa, P. O. Deviveiros, A. Dewhurst, S. Dhaliwal, F. A. Di Bello, A. Di Ciaccio, L. Di Ciaccio, W. K. Di Clemente, C. Di Donato, A. Di Girolamo, B. Di Micco, R. Di Nardo, K. F. Di Petrillo, A. Di Simone, R. Di Sipio, D. Di Valentino, C. Diaconu, M. Diamond, F. A. Dias, T. Dias Do Vale, M. A. Diaz, J. Dickinson, E. B. Diehl, J. Dietrich, S. Díez Cornell, A. Dimitrievska, J. Dingfelder, F. Dittus, F. Djama, T. Djobava, J. I. Djuvsland, M. A. B. Do Vale, M. Dobre, D. Dodsworth, C. Doglioni, J. Dolejsi, Z. Dolezal, M. Donadelli, J. Donini, A. D’onofrio, M. D’Onofrio, J. Dopke, A. Doria, M. T. Dova, A. T. Doyle, E. Drechsler, E. Dreyer, T. Dreyer, M. Dris, Y. Du, J. Duarte-Campderros, F. Dubinin, A. Dubreuil, E. Duchovni, G. Duckeck, A. Ducourthial, O. A. Ducu, D. Duda, A. Dudarev, A. C. Dudder, E. M. Duffield, L. Duflot, M. Dührssen, C. Dülsen, M. Dumancic, A. E. Dumitriu, A. K. Duncan, M. Dunford, A. Duperrin, H. Duran Yildiz, M. Düren, A. Durglishvili, D. Duschinger, B. Dutta, D. Duvnjak, M. Dyndal, S. Dysch, B. S. Dziedzic, C. Eckardt, K. M. Ecker, R. C. Edgar, T. Eifert, G. Eigen, K. Einsweiler, T. Ekelof, M. El Kacimi, R. El Kosseifi, V. Ellajosyula, M. Ellert, F. Ellinghaus, A. A. Elliot, N. Ellis, J. Elmsheuser, M. Elsing, D. Emeliyanov, Y. Enari, J. S. Ennis, M. B. Epland, J. Erdmann, A. Ereditato, S. Errede, M. Escalier, C. Escobar, B. Esposito, O. Estrada Pastor, A. I. Etienvre, E. Etzion, H. Evans, A. Ezhilov, M. Ezzi, F. Fabbri, L. Fabbri, V. Fabiani, G. Facini, R. M. Faisca Rodrigues Pereira, R. M. Fakhrutdinov, S. Falciano, P. J. Falke, S. Falke, J. Faltova, Y. Fang, M. Fanti, A. Farbin, A. Farilla, E. M. Farina, T. Farooque, S. Farrell, S. M. Farrington, P. Farthouat, F. Fassi, P. Fassnacht, D. Fassouliotis, M. Faucci Giannelli, A. Favareto, W. J. Fawcett, L. Fayard, O. L. Fedin, W. Fedorko, M. Feickert, S. Feigl, L. Feligioni, C. Feng, E. J. Feng, M. Feng, M. J. Fenton, A. B. Fenyuk, L. Feremenga, J. Ferrando, A. Ferrari, P. Ferrari, R. Ferrari, D. E. Ferreira de Lima, A. Ferrer, D. Ferrere, C. Ferretti, F. Fiedler, A. Filipčič, F. Filthaut, K. D. Finelli, M. C. N. Fiolhais, L. Fiorini, C. Fischer, W. C. Fisher, N. Flaschel, I. Fleck, P. Fleischmann, R. R. M. Fletcher, T. Flick, B. M. Flierl, L. M. Flores, L. R. Flores Castillo, N. Fomin, G. T. Forcolin, A. Formica, F. A. Förster, A. C. Forti, A. G. Foster, D. Fournier, H. Fox, S. Fracchia, P. Francavilla, M. Franchini, S. Franchino, D. Francis, L. Franconi, M. Franklin, M. Frate, M. Fraternali, D. Freeborn, S. M. Fressard-Batraneanu, B. Freund, W. S. Freund, D. Froidevaux, J. A. Frost, C. Fukunaga, T. Fusayasu, J. Fuster, O. Gabizon, A. Gabrielli, A. Gabrielli, G. P. Gach, S. Gadatsch, P. Gadow, G. Gagliardi, L. G. Gagnon, C. Galea, B. Galhardo, E. J. Gallas, B. J. Gallop, P. Gallus, G. Galster, R. Gamboa Goni, K. K. Gan, S. Ganguly, Y. Gao, Y. S. Gao, C. García, J. E. García Navarro, J. A. García Pascual, M. Garcia-Sciveres, R. W. Gardner, N. Garelli, V. Garonne, K. Gasnikova, A. Gaudiello, G. Gaudio, I. L. Gavrilenko, A. Gavrilyuk, C. Gay, G. Gaycken, E. N. Gazis, C. N. P. Gee, J. Geisen, M. Geisen, M. P. Geisler, K. Gellerstedt, C. Gemme, M. H. Genest, C. Geng, S. Gentile, C. Gentsos, S. George, D. Gerbaudo, G. Gessner, S. Ghasemi, M. Ghasemi Bostanabad, M. Ghneimat, B. Giacobbe, S. Giagu, N. Giangiacomi, P. Giannetti, S. M. Gibson, M. Gignac, D. Gillberg, G. Gilles, D. M. Gingrich, M. P. Giordani, F. M. Giorgi, P. F. Giraud, P. Giromini, G. Giugliarelli, D. Giugni, F. Giuli, M. Giulini, S. Gkaitatzis, I. Gkialas, E. L. Gkougkousis, P. Gkountoumis, L. K. Gladilin, C. Glasman, J. Glatzer, P. C. F. Glaysher, A. Glazov, M. Goblirsch-Kolb, J. Godlewski, S. Goldfarb, T. Golling, D. Golubkov, A. Gomes, R. Goncalves Gama, R. Gonçalo, G. Gonella, L. Gonella, A. Gongadze, F. Gonnella, J. L. Gonski, S. González de la Hoz, S. Gonzalez-Sevilla, L. Goossens, P. A. Gorbounov, H. A. Gordon, B. Gorini, E. Gorini, A. Gorišek, A. T. Goshaw, C. Gössling, M. I. Gostkin, C. A. Gottardo, C. R. Goudet, D. Goujdami, A. G. Goussiou, N. Govender, C. Goy, E. Gozani, I. Grabowska-Bold, P. O. J. Gradin, E. C. Graham, J. Gramling, E. Gramstad, S. Grancagnolo, V. Gratchev, P. M. Gravila, C. Gray, H. M. Gray, Z. D. Greenwood, C. Grefe, K. Gregersen, I. M. Gregor, P. Grenier, K. Grevtsov, J. Griffiths, A. A. Grillo, K. Grimm, S. Grinstein, Ph. Gris, J.-F. Grivaz, S. Groh, E. Gross, J. Grosse-Knetter, G. C. Grossi, Z. J. Grout, C. Grud, A. Grummer, L. Guan, W. Guan, J. Guenther, A. Guerguichon, F. Guescini, D. Guest, R. Gugel, B. Gui, T. Guillemin, S. Guindon, U. Gul, C. Gumpert, J. Guo, W. Guo, Y. Guo, Z. Guo, R. Gupta, S. Gurbuz, G. Gustavino, B. J. Gutelman, P. Gutierrez, C. Gutschow, C. Guyot, M. P. Guzik, C. Gwenlan, C. B. Gwilliam, A. Haas, C. Haber, H. K. Hadavand, N. Haddad, A. Hadef, S. Hageböck, M. Hagihara, H. Hakobyan, M. Haleem, J. Haley, G. Halladjian, G. D. Hallewell, K. Hamacher, P. Hamal, K. Hamano, A. Hamilton, G. N. Hamity, K. Han, L. Han, S. Han, K. Hanagaki, M. Hance, D. M. Handl, B. Haney, R. Hankache, P. Hanke, E. Hansen, J. B. Hansen, J. D. Hansen, M. C. Hansen, P. H. Hansen, K. Hara, A. S. Hard, T. Harenberg, S. Harkusha, P. F. Harrison, N. M. Hartmann, Y. Hasegawa, A. Hasib, S. Hassani, S. Haug, R. Hauser, L. Hauswald, L. B. Havener, M. Havranek, C. M. Hawkes, R. J. Hawkings, D. Hayden, C. Hayes, C. P. Hays, J. M. Hays, H. S. Hayward, S. J. Haywood, M. P. Heath, V. Hedberg, L. Heelan, S. Heer, K. K. Heidegger, J. Heilman, S. Heim, T. Heim, B. Heinemann, J. J. Heinrich, L. Heinrich, C. Heinz, J. Hejbal, L. Helary, A. Held, S. Hellesund, S. Hellman, C. Helsens, R. C. W. Henderson, Y. Heng, S. Henkelmann, A. M. Henriques Correia, G. H. Herbert, H. Herde, V. Herget, Y. Hernández Jiménez, H. Herr, G. Herten, R. Hertenberger, L. Hervas, T. C. Herwig, G. G. Hesketh, N. P. Hessey, J. W. Hetherly, S. Higashino, E. Higón-Rodriguez, K. Hildebrand, E. Hill, J. C. Hill, K. K. Hill, K. H. Hiller, S. J. Hillier, M. Hils, I. Hinchliffe, M. Hirose, D. Hirschbuehl, B. Hiti, O. Hladik, D. R. Hlaluku, X. Hoad, J. Hobbs, N. Hod, M. C. Hodgkinson, A. Hoecker, M. R. Hoeferkamp, F. Hoenig, D. Hohn, D. Hohov, T. R. Holmes, M. Holzbock, M. Homann, S. Honda, T. Honda, T. M. Hong, A. Hönle, B. H. Hooberman, W. H. Hopkins, Y. Horii, P. Horn, A. J. Horton, L. A. Horyn, J-Y. Hostachy, A. Hostiuc, S. Hou, A. Hoummada, J. Howarth, J. Hoya, M. Hrabovsky, J. Hrdinka, I. Hristova, J. Hrivnac, A. Hrynevich, T. Hryn’ova, P. J. Hsu, S.-C. Hsu, Q. Hu, S. Hu, Y. Huang, Z. Hubacek, F. Hubaut, M. Huebner, F. Huegging, T. B. Huffman, E. W. Hughes, M. Huhtinen, R. F. H. Hunter, P. Huo, A. M. Hupe, N. Huseynov, J. Huston, J. Huth, R. Hyneman, G. Iacobucci, G. Iakovidis, I. Ibragimov, L. Iconomidou-Fayard, Z. Idrissi, P. Iengo, R. Ignazzi, O. Igonkina, R. Iguchi, T. Iizawa, Y. Ikegami, M. Ikeno, D. Iliadis, N. Ilic, F. Iltzsche, G. Introzzi, M. Iodice, K. Iordanidou, V. Ippolito, M. F. Isacson, N. Ishijima, M. Ishino, M. Ishitsuka, C. Issever, S. Istin, F. Ito, J. M. Iturbe Ponce, R. Iuppa, A. Ivina, H. Iwasaki, J. M. Izen, V. Izzo, S. Jabbar, P. Jacka, P. Jackson, R. M. Jacobs, V. Jain, G. Jäkel, K. B. Jakobi, K. Jakobs, S. Jakobsen, T. Jakoubek, D. O. Jamin, D. K. Jana, R. Jansky, J. Janssen, M. Janus, P. A. Janus, G. Jarlskog, N. Javadov, T. Javůrek, M. Javurkova, F. Jeanneau, L. Jeanty, J. Jejelava, A. Jelinskas, P. Jenni, J. Jeong, C. Jeske, S. Jézéquel, H. Ji, J. Jia, H. Jiang, Y. Jiang, Z. Jiang, S. Jiggins, F. A. Jimenez Morales, J. Jimenez Pena, S. Jin, A. Jinaru, O. Jinnouchi, H. Jivan, P. Johansson, K. A. Johns, C. A. Johnson, W. J. Johnson, K. Jon-And, R. W. L. Jones, S. D. Jones, S. Jones, T. J. Jones, J. Jongmanns, P. M. Jorge, J. Jovicevic, X. Ju, J. J. Junggeburth, A. Juste Rozas, A. Kaczmarska, M. Kado, H. Kagan, M. Kagan, T. Kaji, E. Kajomovitz, C. W. Kalderon, A. Kaluza, S. Kama, A. Kamenshchikov, L. Kanjir, Y. Kano, V. A. Kantserov, J. Kanzaki, B. Kaplan, L. S. Kaplan, D. Kar, M. J. Kareem, E. Karentzos, S. N. Karpov, Z. M. Karpova, V. Kartvelishvili, A. N. Karyukhin, K. Kasahara, L. Kashif, R. D. Kass, A. Kastanas, Y. Kataoka, C. Kato, J. Katzy, K. Kawade, K. Kawagoe, T. Kawamoto, G. Kawamura, E. F. Kay, V. F. Kazanin, R. Keeler, R. Kehoe, J. S. Keller, E. Kellermann, J. J. Kempster, J Kendrick, O. Kepka, S. Kersten, B. P. Kerševan, R. A. Keyes, M. Khader, F. Khalil-Zada, A. Khanov, A. G. Kharlamov, T. Kharlamova, A. Khodinov, T. J. Khoo, E. Khramov, J. Khubua, S. Kido, M. Kiehn, C. R. Kilby, S. H. Kim, Y. K. Kim, N. Kimura, O. M. Kind, B. T. King, D. Kirchmeier, J. Kirk, A. E. Kiryunin, T. Kishimoto, D. Kisielewska, V. Kitali, O. Kivernyk, E. Kladiva, T. Klapdor-Kleingrothaus, M. H. Klein, M. Klein, U. Klein, K. Kleinknecht, P. Klimek, A. Klimentov, R. Klingenberg, T. Klingl, T. Klioutchnikova, F. F. Klitzner, P. Kluit, S. Kluth, E. Kneringer, E. B. F. G. Knoops, A. Knue, A. Kobayashi, D. Kobayashi, T. Kobayashi, M. Kobel, M. Kocian, P. Kodys, T. Koffas, E. Koffeman, N. M. Köhler, T. Koi, M. Kolb, I. Koletsou, T. Kondo, N. Kondrashova, K. Köneke, A. C. König, T. Kono, R. Konoplich, V. Konstantinides, N. Konstantinidis, B. Konya, R. Kopeliansky, S. Koperny, K. Korcyl, K. Kordas, A. Korn, I. Korolkov, E. V. Korolkova, O. Kortner, S. Kortner, T. Kosek, V. V. Kostyukhin, A. Kotwal, A. Koulouris, A. Kourkoumeli-Charalampidi, C. Kourkoumelis, E. Kourlitis, V. Kouskoura, A. B. Kowalewska, R. Kowalewski, T. Z. Kowalski, C. Kozakai, W. Kozanecki, A. S. Kozhin, V. A. Kramarenko, G. Kramberger, D. Krasnopevtsev, M. W. Krasny, A. Krasznahorkay, D. Krauss, J. A. Kremer, J. Kretzschmar, P. Krieger, K. Krizka, K. Kroeninger, H. Kroha, J. Kroll, J. Kroll, J. Krstic, U. Kruchonak, H. Krüger, N. Krumnack, M. C. Kruse, T. Kubota, S. Kuday, J. T. Kuechler, S. Kuehn, A. Kugel, F. Kuger, T. Kuhl, V. Kukhtin, R. Kukla, Y. Kulchitsky, S. Kuleshov, Y. P. Kulinich, M. Kuna, T. Kunigo, A. Kupco, T. Kupfer, O. Kuprash, H. Kurashige, L. L. Kurchaninov, Y. A. Kurochkin, M. G. Kurth, E. S. Kuwertz, M. Kuze, J. Kvita, T. Kwan, A. La Rosa, J. L. La Rosa Navarro, L. La Rotonda, F. La Ruffa, C. Lacasta, F. Lacava, J. Lacey, D. P. J. Lack, H. Lacker, D. Lacour, E. Ladygin, R. Lafaye, B. Laforge, T. Lagouri, S. Lai, S. Lammers, W. Lampl, E. Lançon, U. Landgraf, M. P. J. Landon, M. C. Lanfermann, V. S. Lang, J. C. Lange, R. J. Langenberg, A. J. Lankford, F. Lanni, K. Lantzsch, A. Lanza, A. Lapertosa, S. Laplace, J. F. Laporte, T. Lari, F. Lasagni Manghi, M. Lassnig, T. S. Lau, A. Laudrain, A. T. Law, P. Laycock, M. Lazzaroni, B. Le, O. Le Dortz, E. Le Guirriec, E. P. Le Quilleuc, M. LeBlanc, T. LeCompte, F. Ledroit-Guillon, C. A. Lee, G. R. Lee, L. Lee, S. C. Lee, B. Lefebvre, M. Lefebvre, F. Legger, C. Leggett, G. Lehmann Miotto, W. A. Leight, A. Leisos, M. A. L. Leite, R. Leitner, D. Lellouch, B. Lemmer, K. J. C. Leney, T. Lenz, B. Lenzi, R. Leone, S. Leone, C. Leonidopoulos, G. Lerner, C. Leroy, R. Les, A. A. J. Lesage, C. G. Lester, M. Levchenko, J. Levêque, D. Levin, L. J. Levinson, D. Lewis, B. Li, C-Q. Li, H. Li, L. Li, Q. Li, Q. Y. Li, S. Li, X. Li, Y. Li, Z. Liang, B. Liberti, A. Liblong, K. Lie, S. Liem, A. Limosani, C. Y. Lin, K. Lin, T. H. Lin, R. A. Linck, B. E. Lindquist, A. L. Lionti, E. Lipeles, A. Lipniacka, M. Lisovyi, T. M. Liss, A. Lister, A. M. Litke, J. D. Little, B. Liu, B. L Liu, H. B. Liu, H. Liu, J. B. Liu, J. K. K. Liu, K. Liu, M. Liu, P. Liu, Y. Liu, Y. L. Liu, Y. W. Liu, M. Livan, A. Lleres, J. Llorente Merino, S. L. Lloyd, C. Y. Lo, F. Lo Sterzo, E. M. Lobodzinska, P. Loch, F. K. Loebinger, K. M. Loew, T. Lohse, K. Lohwasser, M. Lokajicek, B. A. Long, J. D. Long, R. E. Long, L. Longo, K. A. Looper, J. A. Lopez, I. Lopez Paz, A. Lopez Solis, J. Lorenz, N. Lorenzo Martinez, M. Losada, P. J. Lösel, A. Lösle, X. Lou, X. Lou, A. Lounis, J. Love, P. A. Love, J. J. Lozano Bahilo, H. Lu, N. Lu, Y. J. Lu, H. J. Lubatti, C. Luci, A. Lucotte, C. Luedtke, F. Luehring, I. Luise, W. Lukas, L. Luminari, B. Lund-Jensen, M. S. Lutz, P. M. Luzi, D. Lynn, R. Lysak, E. Lytken, F. Lyu, V. Lyubushkin, H. Ma, L. L. Ma, Y. Ma, G. Maccarrone, A. Macchiolo, C. M. Macdonald, J. Machado Miguens, D. Madaffari, R. Madar, W. F. Mader, A. Madsen, N. Madysa, J. Maeda, S. Maeland, T. Maeno, A. S. Maevskiy, V. Magerl, C. Maidantchik, T. Maier, A. Maio, O. Majersky, S. Majewski, Y. Makida, N. Makovec, B. Malaescu, Pa. Malecki, V. P. Maleev, F. Malek, U. Mallik, D. Malon, C. Malone, S. Maltezos, S. Malyukov, J. Mamuzic, G. Mancini, I. Mandić, J. Maneira, L. Manhaes de Andrade Filho, J. Manjarres Ramos, K. H. Mankinen, A. Mann, A. Manousos, B. Mansoulie, J. D. Mansour, M. Mantoani, S. Manzoni, G. Marceca, L. March, L. Marchese, G. Marchiori, M. Marcisovsky, C. A. Marin Tobon, M. Marjanovic, D. E. Marley, F. Marroquim, Z. Marshall, M. U. F Martensson, S. Marti-Garcia, C. B. Martin, T. A. Martin, V. J. Martin, B. Martin dit Latour, M. Martinez, V. I. Martinez Outschoorn, S. Martin-Haugh, V. S. Martoiu, A. C. Martyniuk, A. Marzin, L. Masetti, T. Mashimo, R. Mashinistov, J. Masik, A. L. Maslennikov, L. H. Mason, L. Massa, P. Mastrandrea, A. Mastroberardino, T. Masubuchi, P. Mättig, J. Maurer, B. Maček, S. J. Maxfield, D. A. Maximov, R. Mazini, I. Maznas, S. M. Mazza, N. C. Mc Fadden, G. Mc Goldrick, S. P. Mc Kee, A. McCarn, T. G. McCarthy, L. I. McClymont, E. F. McDonald, J. A. Mcfayden, G. Mchedlidze, M. A. McKay, K. D. McLean, S. J. McMahon, P. C. McNamara, C. J. McNicol, R. A. McPherson, J. E. Mdhluli, Z. A. Meadows, S. Meehan, T. M. Megy, S. Mehlhase, A. Mehta, T. Meideck, B. Meirose, D. Melini, B. R. Mellado Garcia, J. D. Mellenthin, M. Melo, F. Meloni, A. Melzer, S. B. Menary, E. D. Mendes Gouveia, L. Meng, X. T. Meng, A. Mengarelli, S. Menke, E. Meoni, S. Mergelmeyer, C. Merlassino, P. Mermod, L. Merola, C. Meroni, F. S. Merritt, A. Messina, J. Metcalfe, A. S. Mete, C. Meyer, J. Meyer, J-P. Meyer, H. Meyer Zu Theenhausen, F. Miano, R. P. Middleton, L. Mijović, G. Mikenberg, M. Mikestikova, M. Mikuž, M. Milesi, A. Milic, D. A. Millar, D. W. Miller, A. Milov, D. A. Milstead, A. A. Minaenko, I. A. Minashvili, A. I. Mincer, B. Mindur, M. Mineev, Y. Minegishi, Y. Ming, L. M. Mir, A. Mirto, K. P. Mistry, T. Mitani, J. Mitrevski, V. A. Mitsou, A. Miucci, P. S. Miyagawa, A. Mizukami, J. U. Mjörnmark, T. Mkrtchyan, M. Mlynarikova, T. Moa, K. Mochizuki, P. Mogg, S. Mohapatra, S. Molander, R. Moles-Valls, M. C. Mondragon, K. Mönig, J. Monk, E. Monnier, A. Montalbano, J. Montejo Berlingen, F. Monticelli, S. Monzani, R. W. Moore, N. Morange, D. Moreno, M. Moreno Llácer, P. Morettini, M. Morgenstern, S. Morgenstern, D. Mori, T. Mori, M. Morii, M. Morinaga, V. Morisbak, A. K. Morley, G. Mornacchi, A. P. Morris, J. D. Morris, L. Morvaj, P. Moschovakos, M. Mosidze, H. J. Moss, J. Moss, K. Motohashi, R. Mount, E. Mountricha, E. J. W. Moyse, S. Muanza, F. Mueller, J. Mueller, R. S. P. Mueller, D. Muenstermann, P. Mullen, G. A. Mullier, F. J. Munoz Sanchez, P. Murin, W. J. Murray, A. Murrone, M. Muškinja, C. Mwewa, A. G. Myagkov, J. Myers, M. Myska, B. P. Nachman, O. Nackenhorst, K. Nagai, K. Nagano, Y. Nagasaka, K. Nagata, M. Nagel, E. Nagy, A. M. Nairz, Y. Nakahama, K. Nakamura, T. Nakamura, I. Nakano, H. Nanjo, F. Napolitano, R. F. Naranjo Garcia, R. Narayan, D. I. Narrias Villar, I. Naryshkin, T. Naumann, G. Navarro, R. Nayyar, H. A. Neal, P. Y. Nechaeva, T. J. Neep, A. Negri, M. Negrini, S. Nektarijevic, C. Nellist, M. E. Nelson, S. Nemecek, P. Nemethy, M. Nessi, M. S. Neubauer, M. Neumann, P. R. Newman, T. Y. Ng, Y. S. Ng, H. D. N. Nguyen, T. Nguyen Manh, E. Nibigira, R. B. Nickerson, R. Nicolaidou, J. Nielsen, N. Nikiforou, V. Nikolaenko, I. Nikolic-Audit, K. Nikolopoulos, P. Nilsson, Y. Ninomiya, A. Nisati, N. Nishu, R. Nisius, I. Nitsche, T. Nitta, T. Nobe, Y. Noguchi, M. Nomachi, I. Nomidis, M. A. Nomura, T. Nooney, M. Nordberg, N. Norjoharuddeen, T. Novak, O. Novgorodova, R. Novotny, M. Nozaki, L. Nozka, K. Ntekas, E. Nurse, F. Nuti, F. G. Oakham, H. Oberlack, T. Obermann, J. Ocariz, A. Ochi, I. Ochoa, J. P. Ochoa-Ricoux, K. O’Connor, S. Oda, S. Odaka, A. Oh, S. H. Oh, C. C. Ohm, H. Oide, H. Okawa, Y. Okazaki, Y. Okumura, T. Okuyama, A. Olariu, L. F. Oleiro Seabra, S. A. Olivares Pino, D. Oliveira Damazio, J. L. Oliver, M. J. R. Olsson, A. Olszewski, J. Olszowska, D. C. O’Neil, A. Onofre, K. Onogi, P. U. E. Onyisi, H. Oppen, M. J. Oreglia, Y. Oren, D. Orestano, E. C. Orgill, N. Orlando, A. A. O’Rourke, R. S. Orr, B. Osculati, V. O’Shea, R. Ospanov, G. Otero y Garzon, H. Otono, M. Ouchrif, F. Ould-Saada, A. Ouraou, Q. Ouyang, M. Owen, R. E. Owen, V. E. Ozcan, N. Ozturk, J. Pacalt, H. A. Pacey, K. Pachal, A. Pacheco Pages, L. Pacheco Rodriguez, C. Padilla Aranda, S. Pagan Griso, M. Paganini, G. Palacino, S. Palazzo, S. Palestini, M. Palka, D. Pallin, I. Panagoulias, C. E. Pandini, J. G. Panduro Vazquez, P. Pani, G. Panizzo, L. Paolozzi, T. D. Papadopoulou, K. Papageorgiou, A. Paramonov, D. Paredes Hernandez, B. Parida, A. J. Parker, K. A. Parker, M. A. Parker, F. Parodi, J. A. Parsons, U. Parzefall, V. R. Pascuzzi, J. M. P. Pasner, E. Pasqualucci, S. Passaggio, F. Pastore, P. Pasuwan, S. Pataraia, J. R. Pater, A. Pathak, T. Pauly, B. Pearson, M. Pedersen, L. Pedraza Diaz, S. Pedraza Lopez, R. Pedro, S. V. Peleganchuk, O. Penc, C. Peng, H. Peng, B. S. Peralva, M. M. Perego, A. P. Pereira Peixoto, D. V. Perepelitsa, F. Peri, L. Perini, H. Pernegger, S. Perrella, V. D. Peshekhonov, K. Peters, R. F. Y. Peters, B. A. Petersen, T. C. Petersen, E. Petit, A. Petridis, C. Petridou, P. Petroff, E. Petrolo, M. Petrov, F. Petrucci, M. Pettee, N. E. Pettersson, A. Peyaud, R. Pezoa, T. Pham, F. H. Phillips, P. W. Phillips, G. Piacquadio, E. Pianori, A. Picazio, M. A. Pickering, R. Piegaia, J. E. Pilcher, A. D. Pilkington, M. Pinamonti, J. L. Pinfold, M. Pitt, M.-A. Pleier, V. Pleskot, E. Plotnikova, D. Pluth, P. Podberezko, R. Poettgen, R. Poggi, L. Poggioli, I. Pogrebnyak, D. Pohl, I. Pokharel, G. Polesello, A. Poley, A. Policicchio, R. Polifka, A. Polini, C. S. Pollard, V. Polychronakos, D. Ponomarenko, L. Pontecorvo, G. A. Popeneciu, D. M. Portillo Quintero, S. Pospisil, K. Potamianos, I. N. Potrap, C. J. Potter, H. Potti, T. Poulsen, J. Poveda, T. D. Powell, M. E. Pozo Astigarraga, P. Pralavorio, S. Prell, D. Price, M. Primavera, S. Prince, N. Proklova, K. Prokofiev, F. Prokoshin, S. Protopopescu, J. Proudfoot, M. Przybycien, A. Puri, P. Puzo, J. Qian, Y. Qin, A. Quadt, M. Queitsch-Maitland, A. Qureshi, P. Rados, F. Ragusa, G. Rahal, J. A. Raine, S. Rajagopalan, A. Ramirez Morales, T. Rashid, S. Raspopov, M. G. Ratti, D. M. Rauch, F. Rauscher, S. Rave, B. Ravina, I. Ravinovich, J. H. Rawling, M. Raymond, A. L. Read, N. P. Readioff, M. Reale, D. M. Rebuzzi, A. Redelbach, G. Redlinger, R. Reece, R. G. Reed, K. Reeves, L. Rehnisch, J. Reichert, A. Reiss, C. Rembser, H. Ren, M. Rescigno, S. Resconi, E. D. Resseguie, S. Rettie, E. Reynolds, O. L. Rezanova, P. Reznicek, R. Richter, S. Richter, E. Richter-Was, O. Ricken, M. Ridel, P. Rieck, C. J. Riegel, O. Rifki, M. Rijssenbeek, A. Rimoldi, M. Rimoldi, L. Rinaldi, G. Ripellino, B. Ristić, E. Ritsch, I. Riu, J. C. Rivera Vergara, F. Rizatdinova, E. Rizvi, C. Rizzi, R. T. Roberts, S. H. Robertson, A. Robichaud-Veronneau, D. Robinson, J. E. M. Robinson, A. Robson, E. Rocco, C. Roda, Y. Rodina, S. Rodriguez Bosca, A. Rodriguez Perez, D. Rodriguez Rodriguez, A. M. Rodríguez Vera, S. Roe, C. S. Rogan, O. Røhne, R. Röhrig, C. P. A. Roland, J. Roloff, A. Romaniouk, M. Romano, N. Rompotis, M. Ronzani, L. Roos, S. Rosati, K. Rosbach, P. Rose, N-A. Rosien, E. Rossi, L. P. Rossi, L. Rossini, J. H. N. Rosten, R. Rosten, M. Rotaru, J. Rothberg, D. Rousseau, D. Roy, A. Rozanov, Y. Rozen, X. Ruan, F. Rubbo, F. Rühr, A. Ruiz-Martinez, Z. Rurikova, N. A. Rusakovich, H. L. Russell, J. P. Rutherfoord, N. Ruthmann, E. M. Rüttinger, Y. F. Ryabov, M. Rybar, G. Rybkin, S. Ryu, A. Ryzhov, G. F. Rzehorz, P. Sabatini, G. Sabato, S. Sacerdoti, H. F-W. Sadrozinski, R. Sadykov, F. Safai Tehrani, P. Saha, M. Sahinsoy, A. Sahu, M. Saimpert, M. Saito, T. Saito, H. Sakamoto, A. Sakharov, D. Salamani, G. Salamanna, J. E. Salazar Loyola, D. Salek, P. H. Sales De Bruin, D. Salihagic, A. Salnikov, J. Salt, D. Salvatore, F. Salvatore, A. Salvucci, A. Salzburger, D. Sammel, D. Sampsonidis, D. Sampsonidou, J. Sánchez, A. Sanchez Pineda, H. Sandaker, C. O. Sander, M. Sandhoff, C. Sandoval, D. P. C. Sankey, M. Sannino, Y. Sano, A. Sansoni, C. Santoni, H. Santos, I. Santoyo Castillo, A. Sapronov, J. G. Saraiva, O. Sasaki, K. Sato, E. Sauvan, P. Savard, N. Savic, R. Sawada, C. Sawyer, L. Sawyer, C. Sbarra, A. Sbrizzi, T. Scanlon, J. Schaarschmidt, P. Schacht, B. M. Schachtner, D. Schaefer, L. Schaefer, J. Schaeffer, S. Schaepe, U. Schäfer, A. C. Schaffer, D. Schaile, R. D. Schamberger, N. Scharmberg, V. A. Schegelsky, D. Scheirich, F. Schenck, M. Schernau, C. Schiavi, S. Schier, L. K. Schildgen, Z. M. Schillaci, E. J. Schioppa, M. Schioppa, K. E. Schleicher, S. Schlenker, K. R. Schmidt-Sommerfeld, K. Schmieden, C. Schmitt, S. Schmitt, S. Schmitz, U. Schnoor, L. Schoeffel, A. Schoening, E. Schopf, M. Schott, J. F. P. Schouwenberg, J. Schovancova, S. Schramm, A. Schulte, H-C. Schultz-Coulon, M. Schumacher, B. A. Schumm, Ph. Schune, A. Schwartzman, T. A. Schwarz, H. Schweiger, Ph. Schwemling, R. Schwienhorst, A. Sciandra, G. Sciolla, M. Scornajenghi, F. Scuri, F. Scutti, L. M. Scyboz, J. Searcy, C. D. Sebastiani, P. Seema, S. C. Seidel, A. Seiden, T. Seiss, J. M. Seixas, G. Sekhniaidze, K. Sekhon, S. J. Sekula, N. Semprini-Cesari, S. Sen, S. Senkin, C. Serfon, L. Serin, L. Serkin, M. Sessa, H. Severini, F. Sforza, A. Sfyrla, E. Shabalina, J. D. Shahinian, N. W. Shaikh, L. Y. Shan, R. Shang, J. T. Shank, M. Shapiro, A. S. Sharma, A. Sharma, P. B. Shatalov, K. Shaw, S. M. Shaw, A. Shcherbakova, Y. Shen, N. Sherafati, A. D. Sherman, P. Sherwood, L. Shi, S. Shimizu, C. O. Shimmin, M. Shimojima, I. P. J. Shipsey, S. Shirabe, M. Shiyakova, J. Shlomi, A. Shmeleva, D. Shoaleh Saadi, M. J. Shochet, S. Shojaii, D. R. Shope, S. Shrestha, E. Shulga, P. Sicho, A. M. Sickles, P. E. Sidebo, E. Sideras Haddad, O. Sidiropoulou, A. Sidoti, F. Siegert, Dj. Sijacki, J. Silva, M. Silva, M. V. Silva Oliveira, S. B. Silverstein, L. Simic, S. Simion, E. Simioni, M. Simon, P. Sinervo, N. B. Sinev, M. Sioli, G. Siragusa, I. Siral, S. Yu. Sivoklokov, J. Sjölin, M. B. Skinner, P. Skubic, M. Slater, T. Slavicek, M. Slawinska, K. Sliwa, R. Slovak, V. Smakhtin, B. H. Smart, J. Smiesko, N. Smirnov, S. Yu. Smirnov, Y. Smirnov, L. N. Smirnova, O. Smirnova, J. W. Smith, M. N. K. Smith, R. W. Smith, M. Smizanska, K. Smolek, A. A. Snesarev, I. M. Snyder, S. Snyder, R. Sobie, A. M. Soffa, A. Soffer, A. Søgaard, D. A. Soh, G. Sokhrannyi, C. A. Solans Sanchez, M. Solar, E. Yu. Soldatov, U. Soldevila, A. A. Solodkov, A. Soloshenko, O. V. Solovyanov, V. Solovyev, P. Sommer, H. Son, W. Song, A. Sopczak, F. Sopkova, D. Sosa, C. L. Sotiropoulou, S. Sottocornola, R. Soualah, A. M. Soukharev, D. South, B. C. Sowden, S. Spagnolo, M. Spalla, M. Spangenberg, F. Spanò, D. Sperlich, F. Spettel, T. M. Spieker, R. Spighi, G. Spigo, L. A. Spiller, D. P. Spiteri, M. Spousta, A. Stabile, R. Stamen, S. Stamm, E. Stanecka, R. W. Stanek, C. Stanescu, B. Stanislaus, M. M. Stanitzki, B. Stapf, S. Stapnes, E. A. Starchenko, G. H. Stark, J. Stark, S. H Stark, P. Staroba, P. Starovoitov, S. Stärz, R. Staszewski, M. Stegler, P. Steinberg, B. Stelzer, H. J. Stelzer, O. Stelzer-Chilton, H. Stenzel, T. J. Stevenson, G. A. Stewart, M. C. Stockton, G. Stoicea, P. Stolte, S. Stonjek, A. Straessner, J. Strandberg, S. Strandberg, M. Strauss, P. Strizenec, R. Ströhmer, D. M. Strom, R. Stroynowski, A. Strubig, S. A. Stucci, B. Stugu, J. Stupak, N. A. Styles, D. Su, J. Su, S. Suchek, Y. Sugaya, M. Suk, V. V. Sulin, D. M. S. Sultan, S. Sultansoy, T. Sumida, S. Sun, X. Sun, K. Suruliz, C. J. E. Suster, M. R. Sutton, S. Suzuki, M. Svatos, M. Swiatlowski, S. P. Swift, A. Sydorenko, I. Sykora, T. Sykora, D. Ta, K. Tackmann, J. Taenzer, A. Taffard, R. Tafirout, E. Tahirovic, N. Taiblum, H. Takai, R. Takashima, E. H. Takasugi, K. Takeda, T. Takeshita, Y. Takubo, M. Talby, A. A. Talyshev, J. Tanaka, M. Tanaka, R. Tanaka, R. Tanioka, B. B. Tannenwald, S. Tapia Araya, S. Tapprogge, A. Tarek Abouelfadl Mohamed, S. Tarem, G. Tarna, G. F. Tartarelli, P. Tas, M. Tasevsky, T. Tashiro, E. Tassi, A. Tavares Delgado, Y. Tayalati, A. C. Taylor, A. J. Taylor, G. N. Taylor, P. T. E. Taylor, W. Taylor, A. S. Tee, P. Teixeira-Dias, D. Temple, H. Ten Kate, P. K. Teng, J. J. Teoh, F. Tepel, S. Terada, K. Terashi, J. Terron, S. Terzo, M. Testa, R. J. Teuscher, S. J. Thais, T. Theveneaux-Pelzer, F. Thiele, J. P. Thomas, A. S. Thompson, P. D. Thompson, L. A. Thomsen, E. Thomson, Y. Tian, R. E. Ticse Torres, V. O. Tikhomirov, Yu. A. Tikhonov, S. Timoshenko, P. Tipton, S. Tisserant, K. Todome, S. Todorova-Nova, S. Todt, J. Tojo, S. Tokár, K. Tokushuku, E. Tolley, K. G. Tomiwa, M. Tomoto, L. Tompkins, K. Toms, B. Tong, P. Tornambe, E. Torrence, H. Torres, E. Torró Pastor, C. Tosciri, J. Toth, F. Touchard, D. R. Tovey, C. J. Treado, T. Trefzger, F. Tresoldi, A. Tricoli, I. M. Trigger, S. Trincaz-Duvoid, M. F. Tripiana, W. Trischuk, B. Trocmé, A. Trofymov, C. Troncon, M. Trovatelli, F. Trovato, L. Truong, M. Trzebinski, A. Trzupek, F. Tsai, J. C-L. Tseng, P. V. Tsiareshka, N. Tsirintanis, V. Tsiskaridze, E. G. Tskhadadze, I. I. Tsukerman, V. Tsulaia, S. Tsuno, D. Tsybychev, Y. Tu, A. Tudorache, V. Tudorache, T. T. Tulbure, A. N. Tuna, S. Turchikhin, D. Turgeman, I. Turk Cakir, R. Turra, P. M. Tuts, E. Tzovara, G. Ucchielli, I. Ueda, M. Ughetto, F. Ukegawa, G. Unal, A. Undrus, G. Unel, F. C. Ungaro, Y. Unno, K. Uno, J. Urban, P. Urquijo, P. Urrejola, G. Usai, J. Usui, L. Vacavant, V. Vacek, B. Vachon, K. O. H. Vadla, A. Vaidya, C. Valderanis, E. Valdes Santurio, M. Valente, S. Valentinetti, A. Valero, L. Valéry, R. A. Vallance, A. Vallier, J. A. Valls Ferrer, T. R. Van Daalen, W. Van Den Wollenberg, H. Van der Graaf, P. Van Gemmeren, J. Van Nieuwkoop, I. Van Vulpen, M. C. van Woerden, M. Vanadia, W. Vandelli, A. Vaniachine, P. Vankov, R. Vari, E. W. Varnes, C. Varni, T. Varol, D. Varouchas, A. Vartapetian, K. E. Varvell, G. A. Vasquez, J. G. Vasquez, F. Vazeille, D. Vazquez Furelos, T. Vazquez Schroeder, J. Veatch, V. Vecchio, L. M. Veloce, F. Veloso, S. Veneziano, A. Ventura, M. Venturi, N. Venturi, V. Vercesi, M. Verducci, C. M. Vergel Infante, W. Verkerke, A. T. Vermeulen, J. C. Vermeulen, M. C. Vetterli, N. Viaux Maira, O. Viazlo, I. Vichou, T. Vickey, O. E. Vickey Boeriu, G. H. A. Viehhauser, S. Viel, L. Vigani, M. Villa, M. Villaplana Perez, E. Vilucchi, M. G. Vincter, V. B. Vinogradov, A. Vishwakarma, C. Vittori, I. Vivarelli, S. Vlachos, M. Vogel, P. Vokac, G. Volpi, S. E. von Buddenbrock, E. Von Toerne, V. Vorobel, K. Vorobev, M. Vos, J. H. Vossebeld, N. Vranjes, M. Vranjes Milosavljevic, V. Vrba, M. Vreeswijk, T. Šfiligoj, R. Vuillermet, I. Vukotic, T. Ženiš, L. Živković, P. Wagner, W. Wagner, J. Wagner-Kuhr, H. Wahlberg, S. Wahrmund, K. Wakamiya, V. M. Walbrecht, J. Walder, R. Walker, W. Walkowiak, V. Wallangen, A. M. Wang, C. Wang, F. Wang, H. Wang, H. Wang, J. Wang, J. Wang, P. Wang, Q. Wang, R.-J. Wang, R. Wang, R. Wang, S. M. Wang, W. T. Wang, W. Wang, W. X. Wang, Y. Wang, Z. Wang, C. Wanotayaroj, A. Warburton, C. P. Ward, D. R. Wardrope, A. Washbrook, P. M. Watkins, A. T. Watson, M. F. Watson, G. Watts, S. Watts, B. M. Waugh, A. F. Webb, S. Webb, C. Weber, M. S. Weber, S. A. Weber, S. M. Weber, J. S. Webster, A. R. Weidberg, B. Weinert, J. Weingarten, M. Weirich, C. Weiser, P. S. Wells, T. Wenaus, T. Wengler, S. Wenig, N. Wermes, M. D. Werner, P. Werner, M. Wessels, T. D. Weston, K. Whalen, N. L. Whallon, A. M. Wharton, A. S. White, A. White, M. J. White, R. White, D. Whiteson, B. W. Whitmore, F. J. Wickens, W. Wiedenmann, M. Wielers, C. Wiglesworth, L. A. M. Wiik-Fuchs, A. Wildauer, F. Wilk, H. G. Wilkens, L. J. Wilkins, H. H. Williams, S. Williams, C. Willis, S. Willocq, J. A. Wilson, I. Wingerter-Seez, E. Winkels, F. Winklmeier, O. J. Winston, B. T. Winter, M. Wittgen, M. Wobisch, A. Wolf, T. M. H. Wolf, R. Wolff, M. W. Wolter, H. Wolters, V. W. S. Wong, N. L. Woods, S. D. Worm, B. K. Wosiek, K. W. Woźniak, K. Wraight, M. Wu, S. L. Wu, X. Wu, Y. Wu, T. R. Wyatt, B. M. Wynne, S. Xella, Z. Xi, L. Xia, D. Xu, H. Xu, L. Xu, T. Xu, W. Xu, B. Yabsley, S. Yacoob, K. Yajima, D. P. Yallup, D. Yamaguchi, Y. Yamaguchi, A. Yamamoto, T. Yamanaka, F. Yamane, M. Yamatani, T. Yamazaki, Y. Yamazaki, Z. Yan, H. J. Yang, H. T. Yang, S. Yang, Y. Yang, Z. Yang, W-M. Yao, Y. C. Yap, Y. Yasu, E. Yatsenko, J. Ye, S. Ye, I. Yeletskikh, E. Yigitbasi, E. Yildirim, K. Yorita, K. Yoshihara, C. J. S. Young, C. Young, J. Yu, J. Yu, X. Yue, S. P. Y. Yuen, I. Yusuff, B. Zabinski, G. Zacharis, E. Zaffaroni, R. Zaidan, A. M. Zaitsev, N. Zakharchuk, J. Zalieckas, S. Zambito, D. Zanzi, D. R. Zaripovas, S. V. Zeißner, C. Zeitnitz, G. Zemaityte, J. C. Zeng, Q. Zeng, O. Zenin, D. Zerwas, M. Zgubič, D. F. Zhang, D. Zhang, F. Zhang, G. Zhang, H. Zhang, J. Zhang, L. Zhang, L. Zhang, M. Zhang, P. Zhang, R. Zhang, R. Zhang, X. Zhang, Y. Zhang, Z. Zhang, P. Zhao, X. Zhao, Y. Zhao, Z. Zhao, A. Zhemchugov, B. Zhou, C. Zhou, L. Zhou, M. S. Zhou, M. Zhou, N. Zhou, Y. Zhou, C. G. Zhu, H. L. Zhu, H. Zhu, J. Zhu, Y. Zhu, X. Zhuang, K. Zhukov, V. Zhulanov, A. Zibell, D. Zieminska, N. I. Zimine, S. Zimmermann, Z. Zinonos, M. Zinser, M. Ziolkowski, G. Zobernig, A. Zoccoli, K. Zoch, T. G. Zorbas, R. Zou, M. Zur Nedden, L. Zwalinski, ATLAS Collaboration

**Affiliations:** 1 Department of Physics, University of Adelaide, Adelaide, Australia; 2 Physics Department, SUNY Albany, Albany, NY USA; 3 Department of Physics, University of Alberta, Edmonton, AB Canada; 4 Department of Physics, Ankara University, Ankara, Turkey; 5 Istanbul Aydin University, Istanbul, Turkey; 6 Division of Physics, TOBB University of Economics and Technology, Ankara, Turkey; 7 LAPP, Université Grenoble Alpes, Université Savoie Mont Blanc, CNRS/IN2P3, Annecy, France; 8 High Energy Physics Division, Argonne National Laboratory, Argonne, IL USA; 9 Department of Physics, University of Arizona, Tucson, AZ USA; 10 Department of Physics, University of Texas at Arlington, Arlington, TX USA; 11 Physics Department, National and Kapodistrian University of Athens, Athens, Greece; 12 Physics Department, National Technical University of Athens, Zografou, Greece; 13 Department of Physics, University of Texas at Austin, Austin, TX USA; 14 Bahcesehir University, Faculty of Engineering and Natural Sciences, Istanbul, Turkey; 15 Istanbul Bilgi University, Faculty of Engineering and Natural Sciences, Istanbul, Turkey; 16 Department of Physics, Bogazici University, Istanbul, Turkey; 17 Department of Physics Engineering, Gaziantep University, Gaziantep, Turkey; 18 Institute of Physics, Azerbaijan Academy of Sciences, Baku, Azerbaijan; 19 Institut de Física d’Altes Energies (IFAE), Barcelona Institute of Science and Technology, Barcelona, Spain; 20 Institute of High Energy Physics, Chinese Academy of Sciences, Beijing, China; 21 Physics Department, Tsinghua University, Beijing, China; 22 Department of Physics, Nanjing University, Nanjing, China; 23 University of Chinese Academy of Science (UCAS), Beijing, China; 24 Institute of Physics, University of Belgrade, Belgrade, Serbia; 25 Department for Physics and Technology, University of Bergen, Bergen, Norway; 26 Physics Division, Lawrence Berkeley National Laboratory and University of California, Berkeley, CA USA; 27 Institut für Physik, Humboldt Universität zu Berlin, Berlin, Germany; 28 Albert Einstein Center for Fundamental Physics and Laboratory for High Energy Physics, University of Bern, Bern, Switzerland; 29 School of Physics and Astronomy, University of Birmingham, Birmingham, UK; 30 Centro de Investigaciónes, Universidad Antonio Nariño, Bogota, Colombia; 31 Dipartimento di Fisica e Astronomia, Università di Bologna, Bologna, Italy; 32 INFN Sezione di Bologna, Bologna, Italy; 33 Physikalisches Institut, Universität Bonn, Bonn, Germany; 34 Department of Physics, Boston University, Boston, MA USA; 35 Department of Physics, Brandeis University, Waltham, MA USA; 36 Transilvania University of Brasov, Brasov, Romania; 37 Horia Hulubei National Institute of Physics and Nuclear Engineering, Bucharest, Romania; 38 Department of Physics, Alexandru Ioan Cuza University of Iasi, Iasi, Romania; 39 Physics Department, National Institute for Research and Development of Isotopic and Molecular Technologies, Cluj-Napoca, Romania; 40 University Politehnica Bucharest, Bucharest, Romania; 41 West University in Timisoara, Timisoara, Romania; 42 Faculty of Mathematics, Physics and Informatics, Comenius University, Bratislava, Slovak Republic; 43 Department of Subnuclear Physics, Institute of Experimental Physics of the Slovak Academy of Sciences, Kosice, Slovak Republic; 44 Physics Department, Brookhaven National Laboratory, Upton, NY USA; 45 Departamento de Física, Universidad de Buenos Aires, Buenos Aires, Argentina; 46 Cavendish Laboratory, University of Cambridge, Cambridge, UK; 47 Department of Physics, University of Cape Town, Cape Town, South Africa; 48 Department of Mechanical Engineering Science, University of Johannesburg, Johannesburg, South Africa; 49 School of Physics, University of the Witwatersrand, Johannesburg, South Africa; 50 Department of Physics, Carleton University, Ottawa, ON Canada; 51 Faculté des Sciences Ain Chock, Réseau Universitaire de Physique des Hautes Energies, Université Hassan II, Casablanca, Morocco; 52 Centre National de l’Energie des Sciences Techniques Nucleaires (CNESTEN), Rabat, Morocco; 53 Faculté des Sciences Semlalia, Université Cadi Ayyad, LPHEA-Marrakech, Marrakesh, Morocco; 54 Faculté des Sciences, Université Mohamed Premier and LPTPM, Oujda, Morocco; 55 Faculté des sciences, Université Mohammed V, Rabat, Morocco; 56 CERN, Geneva, Switzerland; 57 Enrico Fermi Institute, University of Chicago, Chicago, IL USA; 58 LPC, Université Clermont Auvergne, CNRS/IN2P3, Clermont-Ferrand, France; 59 Nevis Laboratory, Columbia University, Irvington, NY USA; 60 Niels Bohr Institute, University of Copenhagen, Copenhagen, Denmark; 61 Dipartimento di Fisica, Università della Calabria, Rende, Italy; 62 INFN Gruppo Collegato di Cosenza, Laboratori Nazionali di Frascati, Frascati, Italy; 63 Physics Department, Southern Methodist University, Dallas, TX USA; 64 Physics Department, University of Texas at Dallas, Richardson, TX USA; 65 Department of Physics, Stockholm University, Stockholm, Sweden; 66 Oskar Klein Centre, Stockholm, Sweden; 67 Deutsches Elektronen-Synchrotron DESY, Hamburg and Zeuthen, Hamburg, Germany; 68 Lehrstuhl für Experimentelle Physik IV, Technische Universität Dortmund, Dortmund, Germany; 69 Institut für Kern- und Teilchenphysik, Technische Universität Dresden, Dresden, Germany; 70 Department of Physics, Duke University, Durham, NC USA; 71 SUPA, School of Physics and Astronomy, University of Edinburgh, Edinburgh, UK; 72 INFN e Laboratori Nazionali di Frascati, Frascati, Italy; 73 Physikalisches Institut, Albert-Ludwigs-Universität Freiburg, Freiburg, Germany; 74 II. Physikalisches Institut, Georg-August-Universität Göttingen, Göttingen, Germany; 75 Département de Physique Nucléaire et Corpusculaire, Université de Genève, Geneva, Switzerland; 76 Dipartimento di Fisica, Università di Genova, Genoa, Switzerland; 77 INFN Sezione di Genova, Genoa, Italy; 78 II. Physikalisches Institut, Justus-Liebig-Universität Giessen, Giessen, Germany; 79 SUPA, School of Physics and Astronomy, University of Glasgow, Glasgow, UK; 80 LPSC, Université Grenoble Alpes, CNRS/IN2P3, Grenoble INP, Grenoble, France; 81 Laboratory for Particle Physics and Cosmology, Harvard University, Cambridge, MA USA; 82 Department of Modern Physics and State Key Laboratory of Particle Detection and Electronics, University of Science and Technology of China, Hefei, China; 83 Institute of Frontier and Interdisciplinary Science and Key Laboratory of Particle Physics and Particle Irradiation (MOE), Shandong University, Qingdao, China; 84 School of Physics and Astronomy, Shanghai Jiao Tong University, KLPPAC-MoE, SKLPPC, Shanghai, China; 85 Tsung-Dao Lee Institute, Shanghai, China; 86 Kirchhoff-Institut für Physik, Ruprecht-Karls-Universität Heidelberg, Heidelberg, Germany; 87 Physikalisches Institut, Ruprecht-Karls-Universität Heidelberg, Heidelberg, Germany; 88 Faculty of Applied Information Science, Hiroshima Institute of Technology, Hiroshima, Japan; 89 Department of Physics, Chinese University of Hong Kong, Shatin, N.T. Hong Kong; 90 Department of Physics, University of Hong Kong, Hong Kong, China; 91 Department of Physics and Institute for Advanced Study, Hong Kong University of Science and Technology, Clear Water Bay, Kowloon, Hong Kong, China; 92 Department of Physics, National Tsing Hua University, Hsinchu, Taiwan; 93 Department of Physics, Indiana University, Bloomington, IN USA; 94 INFN Gruppo Collegato di Udine, Sezione di Trieste, Udine, Italy; 95 ICTP, Trieste, Italy; 96 Dipartimento di Chimica, Fisica e Ambiente, Università di Udine, Udine, Italy; 97 INFN Sezione di Lecce, Lecce, Italy; 98 Dipartimento di Matematica e Fisica, Università del Salento, Lecce, Italy; 99 INFN Sezione di Milano, Milan, Italy; 100 Dipartimento di Fisica, Università di Milano, Milan, Italy; 101 INFN Sezione di Napoli, Naples, Italy; 102 Dipartimento di Fisica, Università di Napoli, Naples, Italy; 103 INFN Sezione di Pavia, Pavia, Italy; 104 Dipartimento di Fisica, Università di Pavia, Pavia, Italy; 105 INFN Sezione di Pisa, Pisa, Italy; 106 Dipartimento di Fisica E. Fermi, Università di Pisa, Pisa, Italy; 107 INFN Sezione di Roma, Rome, Italy; 108 Dipartimento di Fisica, Sapienza Università di Roma, Rome, Italy; 109 INFN Sezione di Roma Tor Vergata, Rome, Italy; 110 Dipartimento di Fisica, Università di Roma Tor Vergata, Rome, Italy; 111 INFN Sezione di Roma Tre, Rome, Italy; 112 Dipartimento di Matematica e Fisica, Università Roma Tre, Rome, Italy; 113 INFN-TIFPA, Povo, Italy; 114 Università degli Studi di Trento, Trento, Italy; 115 Institut für Astro- und Teilchenphysik, Leopold-Franzens-Universität, Innsbruck, Austria; 116 University of Iowa, Iowa City, IA USA; 117 Department of Physics and Astronomy, Iowa State University, Ames, IA USA; 118 Joint Institute for Nuclear Research, Dubna, Russia; 119 Departamento de Engenharia Elétrica, Universidade Federal de Juiz de Fora (UFJF), Juiz de Fora, Brazil; 120 Universidade Federal do Rio De Janeiro COPPE/EE/IF, Rio de Janeiro, Brazil; 121 Universidade Federal de São João del Rei (UFSJ), São João del Rei, Brazil; 122 Instituto de Física, Universidade de São Paulo, São Paulo, Brazil; 123 KEK, High Energy Accelerator Research Organization, Tsukuba, Japan; 124 Graduate School of Science, Kobe University, Kobe, Japan; 125 Faculty of Physics and Applied Computer Science, AGH University of Science and Technology, Kraków, Poland; 126 Marian Smoluchowski Institute of Physics, Jagiellonian University, Kraków, Poland; 127 Institute of Nuclear Physics Polish Academy of Sciences, Kraków, Poland; 128 Faculty of Science, Kyoto University, Kyoto, Japan; 129 Kyoto University of Education, Kyoto, Japan; 130 Research Center for Advanced Particle Physics and Department of Physics, Kyushu University, Fukuoka, Japan; 131 Instituto de Física La Plata, Universidad Nacional de La Plata and CONICET, La Plata, Argentina; 132 Physics Department, Lancaster University, Lancaster, UK; 133 Oliver Lodge Laboratory, University of Liverpool, Liverpool, UK; 134 Department of Experimental Particle Physics, Jožef Stefan Institute and Department of Physics, University of Ljubljana, Ljubljana, Slovenia; 135 School of Physics and Astronomy, Queen Mary University of London, London, UK; 136 Department of Physics, Royal Holloway University of London, Egham, UK; 137 Department of Physics and Astronomy, University College London, London, UK; 138 Louisiana Tech University, Ruston, LA USA; 139 Fysiska institutionen, Lunds universitet, Lund, Sweden; 140 Centre de Calcul de l’Institut National de Physique Nucléaire et de Physique des Particules (IN2P3), Villeurbanne, France; 141 Departamento de Física Teorica C-15 and CIAFF, Universidad Autónoma de Madrid, Madrid, Spain; 142 Institut für Physik, Universität Mainz, Mainz, Germany; 143 School of Physics and Astronomy, University of Manchester, Manchester, UK; 144 CPPM, Aix-Marseille Université, CNRS/IN2P3, Marseille, France; 145 Department of Physics, University of Massachusetts, Amherst, MA USA; 146 Department of Physics, McGill University, Montreal, QC Canada; 147 School of Physics, University of Melbourne, Melbourne, VIC Australia; 148 Department of Physics, University of Michigan, Ann Arbor, MI USA; 149 Department of Physics and Astronomy, Michigan State University, East Lansing, MI USA; 150 B.I. Stepanov Institute of Physics, National Academy of Sciences of Belarus, Minsk, Belarus; 151 Research Institute for Nuclear Problems of Byelorussian State University, Minsk, Belarus; 152 Group of Particle Physics, University of Montreal, Montreal, QC Canada; 153 P.N. Lebedev Physical Institute of the Russian Academy of Sciences, Moscow, Russia; 154 Institute for Theoretical and Experimental Physics (ITEP), Moscow, Russia; 155 National Research Nuclear University MEPhI, Moscow, Russia; 156 D.V. Skobeltsyn Institute of Nuclear Physics, M.V. Lomonosov Moscow State University, Moscow, Russia; 157 Fakultät für Physik, Ludwig-Maximilians-Universität München, Munich, Germany; 158 Max-Planck-Institut für Physik (Werner-Heisenberg-Institut), Munich, Germany; 159 Nagasaki Institute of Applied Science, Nagasaki, Japan; 160 Graduate School of Science and Kobayashi-Maskawa Institute, Nagoya University, Nagoya, Japan; 161 Department of Physics and Astronomy, University of New Mexico, Albuquerque, NM USA; 162 Institute for Mathematics, Astrophysics and Particle Physics, Radboud University Nijmegen/Nikhef, Nijmegen, The Netherlands; 163 Nikhef National Institute for Subatomic Physics and University of Amsterdam, Amsterdam, The Netherlands; 164 Department of Physics, Northern Illinois University, DeKalb, IL USA; 165 Budker Institute of Nuclear Physics, SB RAS, Novosibirsk, Russia; 166 Novosibirsk State University, Novosibirsk, Russia; 167 Institute for High Energy Physics of the National Research Centre Kurchatov Institute, Protvino, Russia; 168 Department of Physics, New York University, New York, NY USA; 169 Ohio State University, Columbus, OH USA; 170 Faculty of Science, Okayama University, Okayama, Japan; 171 Homer L. Dodge Department of Physics and Astronomy, University of Oklahoma, Norman, OK USA; 172 Department of Physics, Oklahoma State University, Stillwater, OK USA; 173 Palacký University, RCPTM, Joint Laboratory of Optics, Olomouc, Czech Republic; 174 Center for High Energy Physics, University of Oregon, Eugene, OR USA; 175 LAL, Université Paris-Sud, CNRS/IN2P3, Université Paris-Saclay, Orsay, France; 176 Graduate School of Science, Osaka University, Osaka, Japan; 177 Department of Physics, University of Oslo, Oslo, Norway; 178 Department of Physics, Oxford University, Oxford, UK; 179 LPNHE, Sorbonne Université, Paris Diderot Sorbonne Paris Cité, CNRS/IN2P3, Paris, France; 180 Department of Physics, University of Pennsylvania, Philadelphia, PA USA; 181 Konstantinov Nuclear Physics Institute of National Research Centre “Kurchatov Institute”, PNPI, St. Petersburg, Russia; 182 Department of Physics and Astronomy, University of Pittsburgh, Pittsburgh, PA USA; 183 Laboratório de Instrumentação e Física Experimental de Partículas-LIP, Lisbon, Portugal; 184 Departamento de Física, Faculdade de Ciências, Universidade de Lisboa, Lisbon, Portugal; 185 Departamento de Física, Universidade de Coimbra, Coimbra, Portugal; 186 Centro de Física Nuclear da Universidade de Lisboa, Lisbon, Portugal; 187 Departamento de Física, Universidade do Minho, Braga, Portugal; 188 Departamento de Física Teorica y del Cosmos, Universidad de Granada, Granada, Spain; 189 Dep Física and CEFITEC of Faculdade de Ciências e Tecnologia, Universidade Nova de Lisboa, Caparica, Portugal; 190 Institute of Physics, Academy of Sciences of the Czech Republic, Prague, Czech Republic; 191 Czech Technical University in Prague, Prague, Czech Republic; 192 Faculty of Mathematics and Physics, Charles University, Prague, Czech Republic; 193 Particle Physics Department, Rutherford Appleton Laboratory, Didcot, UK; 194 IRFU, CEA, Université Paris-Saclay, Gif-sur-Yvette, France; 195 Santa Cruz Institute for Particle Physics, University of California Santa Cruz, Santa Cruz, CA USA; 196 Departamento de Física, Pontificia Universidad Católica de Chile, Santiago, Chile; 197 Departamento de Física, Universidad Técnica Federico Santa María, Valparaíso, Chile; 198 Department of Physics, University of Washington, Seattle, WA USA; 199 Department of Physics and Astronomy, University of Sheffield, Sheffield, UK; 200 Department of Physics, Shinshu University, Nagano, Japan; 201 Department Physik, Universität Siegen, Siegen, Germany; 202 Department of Physics, Simon Fraser University, Burnaby, BC Canada; 203 SLAC National Accelerator Laboratory, Stanford, CA USA; 204 Physics Department, Royal Institute of Technology, Stockholm, Sweden; 205 Departments of Physics and Astronomy, Stony Brook University, Stony Brook, NY USA; 206 Department of Physics and Astronomy, University of Sussex, Brighton, UK; 207 School of Physics, University of Sydney, Sydney, Australia; 208 Institute of Physics, Academia Sinica, Taipei, Taiwan; 209 E. Andronikashvili Institute of Physics, Iv. Javakhishvili Tbilisi State University, Tbilisi, Georgia; 210 High Energy Physics Institute, Tbilisi State University, Tbilisi, Georgia; 211 Department of Physics, Technion, Israel Institute of Technology, Haifa, Israel; 212 Raymond and Beverly Sackler School of Physics and Astronomy, Tel Aviv University, Tel Aviv, Israel; 213 Department of Physics, Aristotle University of Thessaloniki, Thessaloniki, Greece; 214 International Center for Elementary Particle Physics and Department of Physics, University of Tokyo, Tokyo, Japan; 215 Graduate School of Science and Technology, Tokyo Metropolitan University, Tokyo, Japan; 216 Department of Physics, Tokyo Institute of Technology, Tokyo, Japan; 217 Tomsk State University, Tomsk, Russia; 218 Department of Physics, University of Toronto, Toronto, ON Canada; 219 TRIUMF, Vancouver, BC Canada; 220 Department of Physics and Astronomy, York University, Toronto, ON Canada; 221 Division of Physics and Tomonaga Center for the History of the Universe, Faculty of Pure and Applied Sciences, University of Tsukuba, Tsukuba, Japan; 222 Department of Physics and Astronomy, Tufts University, Medford, MA USA; 223 Department of Physics and Astronomy, University of California Irvine, Irvine, CA USA; 224 Department of Physics and Astronomy, University of Uppsala, Uppsala, Sweden; 225 Department of Physics, University of Illinois, Urbana, IL USA; 226 Instituto de Física Corpuscular (IFIC), Centro Mixto Universidad de Valencia, CSIC, Valencia, Spain; 227 Department of Physics, University of British Columbia, Vancouver, BC Canada; 228 Department of Physics and Astronomy, University of Victoria, Victoria, BC Canada; 229 Fakultät für Physik und Astronomie, Julius-Maximilians-Universität Würzburg, Würzburg, Germany; 230 Department of Physics, University of Warwick, Coventry, UK; 231 Waseda University, Tokyo, Japan; 232 Department of Particle Physics, Weizmann Institute of Science, Rehovot, Israel; 233 Department of Physics, University of Wisconsin, Madison, WI USA; 234 Fakultät für Mathematik und Naturwissenschaften, Fachgruppe Physik, Bergische Universität Wuppertal, Wuppertal, Germany; 235 Department of Physics, Yale University, New Haven, CT USA; 236 Yerevan Physics Institute, Yerevan, Armenia; 237 CERN, 1211 Geneva 23, Switzerland

## Abstract

Searches for non-resonant and resonant Higgs boson pair production are performed in the $\gamma \gamma WW^*$ channel with the final state of $\gamma \gamma \ell \nu jj$ using 36.1 $\hbox {fb}^{-1}$ of proton–proton collision data recorded at a centre-of-mass energy of $\sqrt{s}=$13 TeV by the ATLAS detector at the Large Hadron Collider. No significant deviation from the Standard Model prediction is observed. A 95% confidence-level observed upper limit of 7.7 pb is set on the cross section for non-resonant production, while the expected limit is 5.4 pb. A search for a narrow-width resonance *X* decaying to a pair of Standard Model Higgs bosons *HH* is performed with the same set of data, and the observed upper limits on $\sigma (pp\rightarrow X)\times B(X\rightarrow HH)$ range between 40.0 and 6.1 pb for masses of the resonance between 260 and 500 GeV, while the expected limits range between 17.6 and 4.4 pb. When deriving the limits above, the Standard Model branching ratios of the $H{\rightarrow }{\gamma \gamma }$ and $H{\rightarrow }WW^*$ are assumed.

## Introduction

A particle consistent with the Standard Model (SM) Higgs boson (*H*) was discovered by both the ATLAS and CMS experiments at the Large Hadron Collider (LHC) in 2012 [1, 2]. Various studies of its properties have been performed [3–7], and no significant deviation from the SM predictions has been found. The SM Higgs boson is a strong probe of physics beyond the SM. This paper documents searches for both non-resonant and resonant production of Higgs boson pairs ($HH$) in the semileptonic $\gamma \gamma WW^*$ final state using 36.1 $\hbox {fb}^{-1}$ of proton–proton ($pp$) collision data recorded by the ATLAS detector at a centre-of-mass energy of $\sqrt{s} = 13\,\hbox {TeV}$. Previous searches for Higgs boson pair production have been performed by both the ATLAS and CMS experiments with data recorded at $\sqrt{s} = 8\,\hbox {TeV}$ in the final states $b\bar{b}b\bar{b}$  [8], $b\bar{b}\gamma \gamma$  [9, 10], $b\bar{b}\tau ^{+}\tau ^{-}$  [11–13] and $\gamma \gamma WW^*$  [11], as well as multi-lepton and multi-photon [14]. The $pp$ collision data at $\sqrt{s} = 13\,\hbox {TeV}$ have been analysed in order to search for Higgs boson pairs in the final states $b\bar{b}b\bar{b}$  [15] and $b\bar{b}WW^*$  [16]. No significant excess was observed compared to the SM prediction. However, it is important to explore the $13\,\hbox {TeV}$ data in the channels that are not covered yet, such as the $\gamma \gamma WW^*$ channel presented in this paper. Although this decay channel is not the most sensitive amongst all possible Higgs boson decays, it relies on the Higgs boson couplings to vector bosons, which are already relatively well measured. Furthermore, this channel will contribute to the final combination of all measurable *HH* decays.

**Fig. 1:**
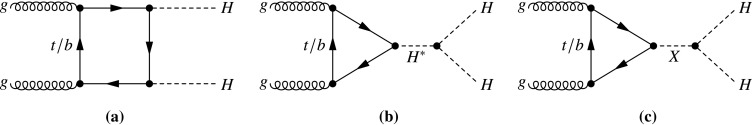
Feynman diagrams for leading-order Higgs boson pair production in the SM through **a** a heavy-quark loop, **b** the Higgs self-coupling, and **c** an intermediate heavy resonance in a BSM scenario. The total SM contribution is the sum of the two modes depicted in **a** and **b**, which have significant destructive interference. Physics beyond the SM can enhance Higgs boson pair production either by modifying the Higgs boson coupling constants from their SM values in **a** and/or **b**, or by an additional s-channel exchange of an intermediate scalar resonance in **c**

The SM prediction of the Higgs boson pair production cross section is several orders of magnitude smaller than the single-Higgs-boson production rate [17], due to additional *ttH* or *HHH* vertices, an additional on-shell Higgs boson that reduces the kinematic phase space, and the fact that the two leading-order (LO) Feynman diagrams have strong destructive interference [18]. In Fig. 1a, the so-called box diagram represents Higgs boson pair production via a heavy-quark loop, where the cross section scales with the squared value of the *ttH* or *bbH* coupling constants. In Fig. 1b, the so-called triangle diagram contributes to Higgs boson pair production via the exchange of a virtual Higgs boson and is the only tree-level diagram sensitive to the Higgs boson self-coupling constant ($\lambda _{HHH}$), the squared value of which scales the cross section.

In many beyond-the-SM (BSM) scenarios, Higgs boson pair production can be enhanced by modifying the *ttH*, *bbH* or $\lambda _{HHH}$ coupling constants from their SM values, reducing the effect of the destructive interference [19] between Fig. 1a and Fig. 1b, or by replacing the virtual Higgs boson with an intermediate scalar resonance, cf. Fig. 1c. Various BSM models with extended Higgs sectors predict a heavy Higgs boson decaying into a pair of Higgs bosons similar to the one in the SM. Such models include the two-Higgs-doublet models (2HDM) [20], the minimal supersymmetric extension of the SM [21], twin Higgs models [22] and composite Higgs models [23, 24]. Heavy resonances, other than heavy Higgs bosons, that can decay into a pair of SM Higgs bosons, are predicted in different models, and could for instance be gravitons [25], radions [26] or stoponium [27].

This paper reports searches for non-resonant and resonant production of pairs of Higgs bosons in the semileptonic $\gamma \gamma WW^*$ final state ($\gamma \gamma \ell \nu jj$), i.e. with two photons, two jets, one charged lepton and a neutrino. This final state benefits from the large branching fraction of $H{\rightarrow }WW^*$  [17], a characteristic signature from two photons and one lepton, as well as the excellent resolution of the diphoton invariant mass $m_{\gamma \gamma }$, which provides good discrimination from a smooth continuum background composed of multi-photon and multi-jet SM processes. Given the expected sensitivity in $13\,\hbox {TeV}$ data, the di-Higgs-boson mass range between 260 and 500 GeV is explored in the search for a scalar resonant Higgs boson pair production.

## The ATLAS detector

The ATLAS experiment [28] is a multipurpose particle detector with a forward-backward symmetric cylindrical geometry and nearly $4\pi$ coverage in solid angle.^1^ It consists of an inner tracking detector (ID) surrounded by a thin superconducting solenoid providing a 2 T axial magnetic field, electromagnetic (EM) and hadronic calorimeters, and a muon spectrometer (MS). The ID covers the pseudorapidity range $|\eta | < 2.5$ and consists of silicon pixel, silicon microstrip, and transition-radiation tracking systems. The innermost pixel layer, the insertable B-layer [29], was installed at a mean radius of 3.3 cm after Run 1, and has been operational since the beginning of Run 2. Lead/liquid-argon (LAr) EM sampling calorimeters with high granularity provide energy measurements of EM showers. A steel/scintillator-tile hadronic calorimeter covers the central pseudorapidity range ($|\eta | < 1.7$). The endcap and forward regions are covered by LAr calorimeters for EM and hadronic energy measurements up to $|\eta | = 4.9$. The MS surrounds the calorimeters and is based on three large air-core toroid superconducting magnets with eight coils each and with bending power in the range 2.0–7.5 T m. It includes a system of fast detectors for triggering purposes and precision tracking chambers. A dedicated two-level trigger system is used to select events [30]. The first-level trigger is implemented in hardware and uses a subset of the detector information to reduce the accepted event rate to at most 100 kHz. This is followed by a software-based trigger level that reduces the accepted event rate to an average of 1 kHz.

## Data and simulated samples

### Data samples

The full set of $pp$ collision data collected during 2015 and 2016 are used in this analysis. The two datasets were recorded at the same centre-of-mass energy $\sqrt{s}$ $=$13 TeV, albeit with different beam conditions. Beam intensities in 2016 were typically higher than in 2015, resulting in a higher instantaneous luminosity and a larger number of $pp$ collisions in each bunch crossing. The integrated luminosity of the combined 2015+2016 dataset used in this analysis is $36.1\pm 0.8\,\hbox {fb}^{-1}$. This dataset were collected in run periods during which all subsystems were operational. The events are collected with a trigger requiring the presence of at least two photons, one with a transverse energy $E_{\text {T}}$ > 35 GeV and the second with $E_{\text {T}}$ > 25 GeV, and the longitudinal and transverse profiles of the EM shower were required to be consistent with those expected for a photon. The corresponding trigger efficiency reaches about 99% for the events that pass the event selection of the analysis.

### Simulated event samples

Simulated Monte Carlo (MC) samples are used to estimate the signal acceptance and study the modelling for both non-resonant SM Higgs boson pair production and resonant BSM Higgs boson pair production. MC samples are also used to estimate the acceptance and study the modelling for SM single-Higgs-boson production processes, and to study the modelling of the SM continuum background from events with multiple photons and jets (Sect. 5), which is the dominant background in the analysis. Eventually, it is estimated by a data-driven method for both its normalisation and shape.

**Table 1:** Simulated signal samples

Processes	Generator	Parton shower	Tune	PDF
Non-resonant	MadGraph5_aMC@NLO 2.2.3	Herwig++	UEEE5	CTEQ6L1
Resonant	MadGraph5_aMC@NLO 2.2.3	Herwig++	UEEE5	CTEQ6L1

The simulated samples for signals are listed in Table 1. The event generator MadGraph5_aMC@NLO 2.2.3 [31] was used for the production of non-resonant [32] and resonant [33] signal MC samples at next-to-leading order (NLO) in QCD, where four values of the resonance mass ($m_{X}$ = 260, 300, 400 and 500 GeV) are considered. The events were generated by a Higgs Effective Field Theory (HEFT) using the MC@NLO method [34] and were reweighted in order to take into account the effects of the finite top-quark mass. The parton shower was implemented using Herwig++ 2.7.1 [35] with a set of tuned underlying-event parameters called the UEEE5 tune^2^ [36], and the parton distribution function (PDF) set CTEQ6L1 [37] was used.

- For non-resonant Higgs boson pair production, the inclusive cross section s are normalised to the SM prediction of 33.41 fb [17, 38], calculated at NNLO in QCD, including resummation of soft-gluon emission at next-to-next-to-leading-logarithmic (NNLL) accuracy, as prescribed by the LHC Higgs Cross Section Working Group [17]. The effect of the finite top-quark mass is also taken into account at NLO [39].
- For resonant Higgs boson pair production, a narrow decay width, which is negligible compared to the experimental mass resolution, is assumed. The interference between non-resonant and resonant Higgs boson pair production is implemented in the generator. The interference is minimal and remains negligible when a narrow decay width is assumed.

**Table 2:** Simulated SM single-Higgs-boson background samples with $m_{H} =$ 125 GeV

Processes	Generators	QCD order	EW order	PDF	Parton shower	Normalisation
ggF	Powheg NNLOPS	NNLO	NLO	PDF4LHC15	Pythia 8.186	N$^3$LO (QCD) + NLO (EW)
VBF	Powheg	NLO	NLO	PDF4LHC15	Pythia 8.186	NNLO (QCD) + NLO (EW)
$W^{+}H$	Powheg MiNLO	NLO	NLO	PDF4LHC15	Pythia 8.186	NNLO (QCD) + NLO (EW)
$W^{-}H$	Powheg MiNLO	NLO	NLO	PDF4LHC15	Pythia 8.186	NNLO (QCD) + NLO (EW)
$q\bar{q}\rightarrow ZH$	Powheg MiNLO	NLO	NLO	PDF4LHC15	Pythia 8.186	NNLO (QCD) + NLO (EW)
*ggZH*	Powheg MiNLO	NLO	NLO	PDF4LHC15	Pythia 8.186	NLO NLL (QCD)
$t\bar{t}H$	MadGraph aMC@NLO	NLO	NLO	NNPDF3.0	Pythia 8.186	NLO (QCD) + NLO (EW)

Table 2 lists the simulated samples for the dominant SM single-Higgs-boson production modes: gluon–gluon fusion (ggF), vector-boson fusion (VBF), associated production with a *W* or *Z* boson ($VH$), and associated production with a pair of top quarks ($t\bar{t}H$). For all these processes, the Pythia 8.186 parton shower is used for the modelling of non-perturbative effects. The AZNLO tune [40] is used in ggF, VBF and $VH$ simulations, while the A14 tune is used in $t\bar{t}H$ simulations.

- **gluon–gluon fusion**:  The ggF production is accurate to NNLO in QCD, using the Powheg method [41] for matching the matrix element with the parton shower, and the MiNLO method [42, 43] to simultaneously achieve NLO accuracy for inclusive Higgs boson production. Furthermore, a reweighting procedure was performed using the HNNLO program [44–46] to achieve full NNLO accuracy [47]. This sample is referred to as NNLOPS. The PDF4LHC15 NLO PDF set [48] was used. The inclusive cross section of the ggF production is normalised to the calculation at next-to-next-to-next-to-leading-order (N$^3$LO) QCD and NLO electroweak (EW) accuracies [49].
- **VBF and** ***VH***:  VBF and $VH$ production was simulated at NLO in QCD with Powheg-Box v2 [41, 50, 51] using the PDF4LHC15 NLO PDF set. The inclusive VBF contribution is normalised to the cross section calculated with NLO QCD and NLO EW corrections [52–54] with an approximate NNLO QCD correction applied [55]. The contributions are normalised to cross section s calculated with NNLO QCD [56] and NLO EW corrections [57] for *WH* and $q\bar{q}\rightarrow ZH$ and at NLO and next-to-leading-logarithm (NLL) accuracy in QCD for $gg\rightarrow ZH$ [58].
- ${\varvec{t}}\bar{{\varvec{t}}}{\varvec{H}}$:  The $t\bar{t}H$ production is simulated using MadGraph5_aMC@NLO 2.2.3 and its inclusive cross section is normalised to a calculation with NLO QCD and NLO EW corrections [59–62].

Processes of continuum backgrounds of multiple photons and jets with either one or zero leptons were simulated with MadGraph5_aMC@NLO 2.2.2, interfaced with the parton shower model in Pythia 8.186.

Multiple $pp$ collisions in each bunch crossing, “pile-up”, were simulated with the soft QCD processes of Pythia 8.186 using the A2 tune [63] and the MSTW2008LO PDF set [64]. An additional event-level reweighting is performed in order to ensure that the distribution of the average number of interactions per bunch crossing matches that occurring in the data used in this analysis. The particles in the final states of the generated processes were passed through either a Geant4  [65] simulation of the ATLAS detector, or through the ATLAS fast simulation framework [66], which has been extensively validated against the Geant4 simulation model. The output from the detector simulation is then analysed using the same reconstruction software as the data. The MC samples for single-Higgs-boson production were simulated with the Geant4 framework, while the other samples used in this analysis were produced with the ATLAS fast simulation framework.

## Object and event selection

The event selection is based on the properties of the visible objects in the final state, which includes one charged lepton (electron or muon), two jets, and two photons. These objects are reconstructed from detector-level objects, such as energy clusters in the EM calorimeter and tracks in the ID, as well as in the MS.

### Object reconstruction

Photon candidates are reconstructed from clusters of energy deposited in the EM calorimeter [67]. If the candidates are matched with a reconstructed conversion vertex or tracks consistent with the hypothesis of a $\gamma {\rightarrow }e^{+}e^{-}$ conversion, they are classified as converted photon candidates.^3^ If the matched track is consistent with the hypothesis of an electron produced in the beam interaction region, they are classified as electron candidates. If the candidates are not matched with a reconstructed conversion vertex or tracks satisfying the conversion requirement, they are classified as unconverted photon candidates. The energy is determined by summing the energies of all cells that belong to the associated cluster [68] and is corrected using a combination of simulation-based and data-driven calibration factors [69] determined from $Z\rightarrow e^+e^-$ events collected in 2015 and 2016. The photon energy resolution in simulation is corrected to match the resolution in data [67]. The reconstructed photon candidates are required to meet “tight” photon identification criteria [68], which are based on the lateral and longitudinal energy profiles of EM showers in the calorimeter. The identification efficiency is measured as a function of the transverse energy of photons ($E_{\text {T}}^{\gamma }$). It ranges from 90 to 98% for converted photons and from 85 to 95% for unconverted photons, in the $E_{\text {T}}^{\gamma }$ interval between 25 and 200 $\text {GeV}$. To suppress the background from jets misidentified as photons, all reconstructed photon candidates are required to meet a set of calorimeter- and track-based isolation criteria [70]. A calorimeter-based isolation variable $E^{\text {iso}}_{\text {T}}$ is defined as the sum of the transverse energies ($E_{\text {T}}$) of all positive-energy topological clusters of calorimeter cells [71] within $\Delta R = 0.2$ of the photon candidate, excluding the energy of the photon candidate itself. The selection applied to the calorimeter-based isolation variable is $E^{\text {iso}}_{\text {T}} < 0.065E_{\text {T}} ^{\gamma }$. A track-based isolation variable $p^{\text {iso}}_{\text {T}}$ is defined as the scalar sum of the transverse momenta ($p_{\text {T}}$) of tracks with $p_{\text {T}}$ > 1 GeV within $\Delta R = 0.2$ of the photon candidate, excluding tracks from photon conversions. The selection on the track-based isolation variable is $p^{\text {iso}}_{\text {T}} < 0.05E_{\text {T}}^{\gamma }$. Only photon candidates with $|\eta |$ < 2.37 are considered, excluding the transition region between the barrel and endcap calorimeters (1.37 < $|\eta |$ < 1.52).

Electron candidates are reconstructed from clusters of energy deposited in the EM calorimeter matched to a track in the inner detector, as described above. A likelihood-based (LH) algorithm is used [72] to perform the electron identification against the background from jets or non-prompt electrons. Electron candidates are identified according to the “medium LH” criteria. Muon candidates are identified by matching a reconstructed ID track with a reconstructed MS track [73]. The identification classifies muon candidates as either “loose” or “medium”, based on the number of hits in the different ID and MS subsystems, and on the significance of the difference $|q/p_{\text {MS}}-q/p_{\text {ID}}|$, where *q* is the charge and *p* is the momentum of the muon candidate, as well as on the energy deposit in the tile hadronic calorimeters. The “medium” candidates are used in the analysis. An efficiency ranging from 84 to 93% as a function of $E_{\text {T}}$ or $p_{\text {T}}$ is achieved in the combined identification and reconstruction of electrons, and 96% (above 98%) in muon identification (reconstruction), in the range where the objects are selected. The electron (muon) is required to pass the “Loose” (“GradientLoose”) isolation criterion based on the sum of $p_{\text {T}}$ of tracks lying within a cone of $\Delta R=$ min (10 $\hbox {GeV/}p_{\text {T}}^{e (\mu )}$, 0.3) and the sum of $E_{\text {T}}$ of topological clusters of calorimeter cells within a cone of $\Delta R=$ 0.2 (0.2) around the electron (muon) candidate, excluding the contributions from the electron (muon) candidate. With these requirements the isolation efficiencies for electrons (muons) are above 99% ($0.057p_{\text {T}}^{\mu }+95.57\%$) [74, 74]. Finally, the electron candidates are required to have $E_{\text {T}} > 10$ GeV and $|\eta |<2.47$, excluding the transition region between the barrel and endcap calorimeters (1.37 < $|\eta |$ < 1.52), whereas the muon candidates are required to have $p_{\text {T}} > 10$ GeV and $|\eta |<2.7$.

Jets are reconstructed via the FastJet package [74] using the anti-$k_{t}$ clustering algorithm [75] with a radius parameter $R=$ 0.4. The jet energies are determined at the EM scale and calibrated using particle-level correction factors based on a combination of simulation and data [76–80]. Jets are required to have $|\eta |$ < 2.5 and $p_{\text {T}}$ > 25 GeV. In addition, a jet-vertex tagging algorithm (JVT)  [81] is applied to jets with $|\eta |$ < 2.4 and $p_{\text {T}}$ < 60 GeV in order to suppress jets originating from pile-up interactions. In this algorithm, a multivariate discriminant based on two track-based variables is constructed to reject pile-up jets while maintaining a high efficiency for the hard-scatter jet independent of the number of primary vertices in the event. The selected jets are classified as $b\text {-jet}$ s using a multivariate technique [82, 83], which takes advantage of the information about secondary vertices, the impact parameters of the associated tracks and the topologies of decays of heavy-flavour hadrons. The *b*-tagging working point is selected to have an efficiency of 70% for a *b*-jet from $t\bar{t}$ decays, with a rejection factor of 12 for jets originating from *c*-quarks (*c*-jets), and of close to 400 for jets initiated by light-flavour quarks or gluons (light-flavour jets).

An overlap removal procedure is performed in the following order to avoid double counting of detector-level objects when reconstructing physics objects. Electrons with $\Delta R(e,\gamma )<$ 0.4 are removed. Jets with $\Delta R(\text {jet},\gamma )<$ 0.4 or $\Delta R(\text {jet},e)<$ 0.2 are removed. Electrons with $\Delta R(e,\text {jet})<$ 0.4 are removed. Muons with $\Delta R(\mu ,\gamma )<$ 0.4 or $\Delta R(\mu ,\text {jet})<$ 0.4 are removed.

### Event selection

**Fig. 2:**
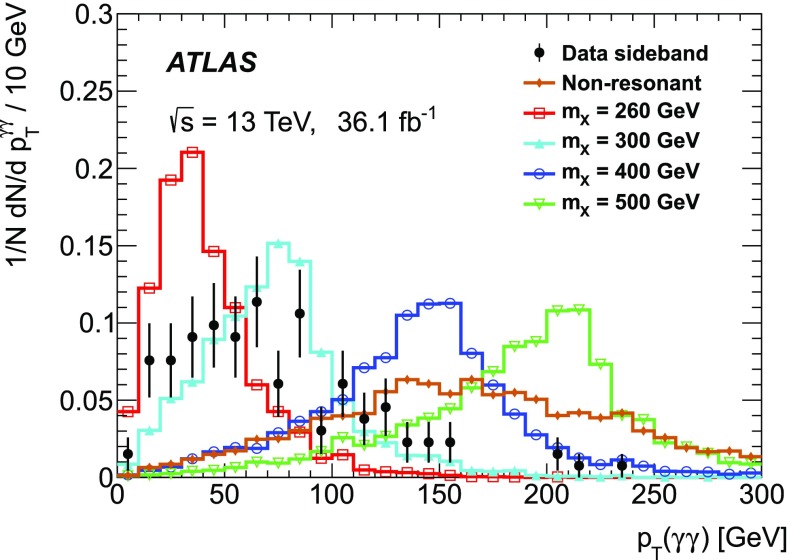
Distributions of the reconstructed transverse momenta of the diphoton system with all event selections, except the $p_{\text {T}} ^{\gamma \gamma }$selection, applied for various signal models, as well as sideband data, normalised to unit area

The events passing the diphoton trigger are required to contain at least two jets, no $b\text {-jet}$, and at least one charged lepton (*e* or $\mu$, but including contributions from fully leptonic $\tau$-lepton decays) in the final state. The two photon candidates with the leading (sub-leading) $E_{\text {T}}$ are required to satisfy $E_{\text {T}} ^{\gamma }/m_{\gamma \gamma }>$ 0.35 (0.25). The $b\text {-jet}$ veto suppresses the $t\bar{t}H$ process. Furthermore, the transverse momentum of the diphoton system ($p_{\text {T}} ^{\gamma \gamma }$) is required to be larger than 100 GeV for maximising the sensitivity and keeping at least 70% of signal events. This requirement suppresses continuum background events when searching for non-resonant Higgs boson pair production, or resonant production with resonance masses of 400 GeV or higher. However, the $p_{\text {T}} ^{\gamma \gamma }$selection is omitted in the search for resonance masses below 400 GeV due to a limited separation between signal and continuum background in this kinematical region, as can be seen in Fig. 2. These final selection criteria, together with a requirement on the invariant diphoton mass of $105\,\hbox {GeV}< m_{\gamma \gamma } < 160\,\hbox {GeV}$, define the event sample on which the signal search is performed for the various assumed signal models. A data “sideband” sample is selected applying the same criteria, but excluding the Higgs mass region $m_{\gamma \gamma }$ 121.7–128.5 GeV, and can be used together with other samples to study the continuum background.

If there were an observable signal, one of the Higgs bosons would be directly visible in the $m_{\gamma \gamma }$ distribution. The combination of two jets and at least one charged lepton would be consistent with $H{\rightarrow }WW^*$ for the other Higgs boson. Its signature would in principle be enhanced by a missing transverse energy ($E_{\text {T}}^{\text {miss}}$) requirement to indicate a neutrino, but a selection on $E_{\text {T}}^{\text {miss}}$ was found not to produce any significant improvement in sensitivity, and so was not applied. The magnitude of $E_{\text {T}}^{\text {miss}}$  [84, 85] is measured from the negative vectorial sum of the transverse momenta of all photon, electron and muon candidates and of all hadronic jets after accounting for overlaps between jets, photons, electrons, and muons, as well as an estimate of soft contributions based on tracks.

After all selections described above, the combined acceptance and selection efficiency for non-resonant production is 8.5%, while it ranges from 6.1 to 10% as a function of the mass of the resonance ($m_X$) from 260 to 500 GeV, as shown in Table 3. The efficiency for the non-resonant Higgs boson pair production is at the same level as the efficiency for the high-mass resonant production, as the Higgs bosons and their decay products tend to exhibit large transverse momenta due to the box diagram shown in Fig. 1a.

**Table 3:** The combined acceptance and efficiency for non-resonant and resonant with different scalar resonance masses $m_{X}$, with and without a $p_{\text {T}} ^{\gamma \gamma }$selection

	No $p_{\text {T}} ^{\gamma \gamma }$selection			$p_{\text {T}} ^{\gamma \gamma }$> 100 GeV		
$m_{X}$ (GeV)	260	300	400	400	500	Non-resonant
Acceptance $\,\times \,$ efficiency (%)	6.1	7.1	9.7	7.8	10	8.5

## Signal and background estimation

A fit to the $m_{\gamma \gamma }$ distribution is performed to extract the signal yield as described in Sect. 7. The shapes of both the signal and background distributions are modelled with analytical functions. For both Higgs boson pair production and single-Higgs-boson processes, the $m_{\gamma \gamma }$ distributions are modelled with double-sided Crystal Ball functions [86]. Their shape parameters are determined by a fit to simulated samples. The single-Higgs-boson contribution is normalised to the SM cross-sections as described in Sect. 3.2. Higgs boson pair production is regarded as a background to the resonant search. Its contribution is also set to the SM prediction of Sect. 3.2.

The continuum background is modelled with an exponential function of a second-order polynomial. Several functional forms were evaluated by fitting the sidebands in data and MC samples under different conditions of photon purity and lepton multiplicity. Photon purity was lowered, compared to the final data selection, by reversing the requirements on photon isolation or identification. For higher photon purity, MC samples with prompt photons were used. The lepton multiplicity was varied to be zero or at least one. For all combinations of conditions, the exponential function with a second-order polynomial gave the best fits, with satisfactory $\chi ^2$, and was chosen to model the continuum background. The shape parameters and normalisation are free to float in the final fit to the data. Since any functional form might introduce spurious signals, this effect is estimated with a sample mixing irreducible prompt-photon background from simulation and reducible backgrounds from data, as described in Sect. 6.

The expected numbers of signal and background events are shown in Table 4 together with the number of events observed in data. Only events within a mass window of $m_{H} \pm 2$ $\sigma _{m_{\gamma \gamma }}$ are reported, where the Higgs boson mass ($m_{H}$) is taken to be 125.09 GeV [87] and the diphoton mass resolution ($\sigma _{m_{\gamma \gamma }}$) is 1.7 GeV and is obtained from simulation. The dominant background is from continuum processes with multiple photons and jets. A small background arises from SM single-Higgs-boson production processes, among which $t\bar{t}H$ and *WH* productions give the leading contributions with, respectively, a fraction of 41.5% (39.2%) and 23.3% (22.5%) of the whole single-Higgs-boson contribution with (without) the $p_{\text {T}} ^{\gamma \gamma }$> 100 GeV selection.

**Table 4:** Numbers of expected and observed events in the $m_{H} \pm 2$ $\sigma _{m_{\gamma \gamma }}$ mass window with or without a $p_{\text {T}} ^{\gamma \gamma }$selection. A cross section of 33.41 fb is assumed for non-resonant Higgs boson pair production when it is considered as a background in resonant searches. The resulting yields are determined from the fit to data by integrating the resulting functional forms over the selected $m_{\gamma \gamma }$ range. The error in each yield includes both the statistical and systematic uncertainties, as discussed in Sect. 6

Process	Number of events	
	No $p_{\text {T}} ^{\gamma \gamma }$selection	$p_{\text {T}} ^{\gamma \gamma }$> 100 GeV
Continuum background	$22\pm 5$	$5.1\pm 2.3$
SM single-Higgs	$1.92\pm 0.15$	$1.0\pm 0.09$
SM di-Higgs	$0.046\pm 0.004$	$0.038\pm 0.004$
Sum of expected background	$24\pm 5$	$6.1\pm 2.3$
Data	33	7

## Systematic uncertainties

### Theoretical uncertainties

Theoretical uncertainties in the prediction of the cross section of single Higgs bosons are estimated from variations of the normalisation and factorisation scales, PDF, and the running QCD coupling constant ($\alpha _\text {S}$) [17]. Among the dominant production modes $t\bar{t}H$ and $VH$, the cross section of $t\bar{t}H$ has the largest uncertainty: up to 9.2% in the scale variations, up to 3.0% in the PDF variations, and 2.0% in the $\alpha _\text {S}$ variations, as prescribed by the LHC Higgs Cross Section Working Group [17].

The theoretical uncertainties in the efficiency times acceptance ($\epsilon \times A$) are estimated from scale, PDF and parton shower variations. The scale uncertainty ranges from 2.1 to 4.1% for resonant production and is 3.4% for non-resonant production. The PDF uncertainty is around 2.5 and 3.0% for the resonant and non-resonant production, respectively. The parton shower uncertainty is estimated by comparing Pythia 8 and Herwig++ as two different shower models, and ranges from 6.0% at $m_{X} = 500$ GeV to 29.6% at $m_{X} = 260$ GeV for resonant production, and is 7.8% for non-resonant production. This uncertainty is large in low-mass resonant production because the jet spectrum at low-$p_\text {T}$ is more susceptible to variations in the parton shower model. Non-resonant Higgs boson pair production is considered as a background in the search for resonant Higgs boson pair production. The scale, PDF, $\alpha _\text {S}$ and HEFT uncertainties in the calculation of the cross section for SM Higgs boson pair production are also taken into account. These values are 6.0%, 2.1%, 2.3%, and 5.0%, respectively, following the recommendations in Ref. [17]. Further uncertainties arising from the $H{\rightarrow }{\gamma \gamma }$ and $H{\rightarrow }WW^*$ branching ratios (*B*) are considered as well. They are 2.1% and 1.5% [17], respectively.

### Modelling uncertainties in the continuum background

The exponential function of a second-order polynomial is determined to provide the simplest and most robust functional form for modelling the continuum background as described in Sect. 5. The uncertainties in the modelling are estimated by fitting a signal-plus-background model to a simulated background-only sample that has such a large number of events that its own statistical uncertainty does not affect the test results. The fitted number of signal events ($n_\text {ss}$) quantifies spurious signal events. The fits are performed with the assumed $m_H$ ranging from 120 to 130 GeV in steps of 0.5 GeV. The maximum value of the fitted signal yields |$n_\text {ss}$| is regarded as a bias in the yields due to the background modelling (the spurious signal), and is, conservatively, taken into account in the fit as the modelling uncertainty. The fitted |$n_\text {ss}$| value reaches as large as 0.46 when not applying the $p_{\text {T}} ^{\gamma \gamma }$ selection, and 0.26 when applying the selection. The simulated background-only samples include the irreducible process of $\gamma \gamma \ell \nu jj$ and the reducible processes represented by events where one or two hadronic jets are misidentified as photons. The reducible processes are modelled by the data events with reversed photon identification or isolation requirements. The two components are combined according to the measured diphoton purity, which is about 88% (90% with $p_{\text {T}} ^{\gamma \gamma }$selection) and normalised according to the number of selected data events.

### Experimental uncertainties

The uncertainty in the measurement of the combined 2015+2016 integrated luminosity is 2.1%. It is derived, following a methodology similar to that detailed in Ref. [88], from a calibration of the luminosity scale using *x*–*y* beam-separation scans performed in August 2015 and May 2016. All processes that are estimated using simulation are affected by the uncertainty in the luminosity measurement.

The efficiency of the diphoton trigger is estimated using bootstrap methods [89] with a systematic uncertainty of 0.4%. The photon identification uncertainty is obtained by varying the data-to-simulation efficiency corrections within their uncertainties, derived from control samples of photons from radiative *Z* boson decays and from inclusive $\gamma$ events, and of electrons from $Z\rightarrow e^+e^-$ decays. A maximal uncertainty of 1.7% in the yields is evaluated in all of the SM single-Higgs-boson, SM di-Higgs-boson and BSM Higgs boson production processes. The photon–track isolation uncertainty is derived from measurements of the uncertainty in the data-to-simulation efficiency corrections using inclusive-photon control samples, while the uncertainty from the calorimeter isolation requirement is evaluated from the difference between applying and not applying corrections derived from inclusive-photon events to the calorimeter isolation variable in the simulation. In general, the overall isolation uncertainty is less than 1%. The uncertainties from the photon energy resolution and scale affect the yields by less than 0.2%. The relevant impact on the shape of the diphoton invariant mass is also considered by introducing variations of the resolution and mean values of the fit function and is estimated using simulation. The photon energy resolution varies the resolution of the $m_{\gamma \gamma }$ shape by 5.2–11.4%, while the photon energy scale affects the mean value by about 0.5%. The jet energy scale (JES) and the corresponding uncertainties are derived from both simulation and in situ calibration using data [77, 90]. This affects the event selection efficiency by 2.4–9.9%, depending on the process. The jet energy resolution (JER) uncertainty is evaluated by smearing jet energies according to the systematic uncertainties of the resolution measurement [80, 91], and its impact on the event selection efficiency ranges from 0.1 to 1.6%. The *b*-tagging uncertainties is derived separately for *b*-jets, *c*-jets and light-flavour jets [82]. Overall, their impact on the yields is not more than 4%. Uncertainties arising from the reconstruction, identification and isolation of both the electron and muon candidates [72, 73], are propagated to the event yield variations, and they are found to have an impact of less than 1%. Finally, the pile-up reweighting procedure, which matches the distribution of the number of interactions per bunch crossing between simulation and data, has associated systematic uncertainties of less than 1%. All experimental uncertainties are correlated among all processes that use simulation to model the yields and the kinematics. A summary of the systematic uncertainties in the expected yields of the di-Higgs-boson and single-Higgs-boson production is presented in Table 5. In the search for non-resonant Higgs boson pair production, SM Higgs boson pair production is considered to be the signal process, while single Higgs boson production is considered to be a background. In the search for the resonant Higgs boson pair production, both SM single Higgs boson production and non-resonant Higgs boson pair productions are considered to be background processes.

**Table 5:** Summary of relative systematic uncertainties, in percent, propagated to the yields for the MC-estimated processes. Entries marked by ‘–’ indicate that the systematic uncertainty is not applicable for the corresponding process. The extrapolation uncertainties in *b*-tagging include two components: one is from the extrapolation to high-$p_{\text {T}}$ ($p_{\text {T}}$ $>300$ GeV) jets and the other one is from extrapolating *c*-jets to $\tau$-jets. The values for resonant production shown here assume $m_X$ = 260 GeV. Several theoretical uncertainties are reported for the cross section ($\sigma$) and the combined efficiency and acceptance ($\epsilon \times A$)

Source of uncertainties	Non-resonant $HH$	$X{\rightarrow }HH$	Single-*H* bkg $p_{\text {T}} ^{\gamma \gamma }$> 100 GeV	Single-*H* bkg No $p_{\text {T}} ^{\gamma \gamma }$selection
Luminosity 2015 + 2016	2.1	2.1	2.1	2.1
Trigger	0.4	0.4	0.4	0.4
Event sample size	1.7	2.2	1.6	1.3
Pile-up reweighting	0.5	0.9	0.7	0.6
Photon				
Identification	1.7	1.4	0.8	0.8
Isolation	0.8	0.7	0.4	0.4
Energy resolution	0.1	0.1	0.2	$< 0.1$
Energy scale	0.2	$< 0.1$	0.2	$< 0.1$
Jet				
Energy scale	4.0	9.9	2.4	2.6
Energy resolution	0.1	1.6	0.5	1.0
*b*-tagging				
*b*-hadron jets	$< 0.1$	$< 0.1$	3.8	3.6
*c*-hadron jets	1.5	1.0	0.7	0.6
Light-flavour jets	0.3	0.3	0.1	0.1
Extrapolation	$< 0.1$	$< 0.1$	0.1	$< 0.1$
Lepton				
Electron	0.5	0.7	0.2	0.2
Muon	0.5	0.7	0.3	0.5
Theory				
PDF on $\sigma$	2.1	–	3.4	3.4
$\alpha _S$ on $\sigma$	2.3	–	1.3	1.3
Scale on $\sigma$	6.0	–	0.9	0.9
HEFT on $\sigma$	5.0	–	–	–
Scale on $\epsilon \times A$	2.8	2.5	–	–
PDF on $\epsilon \times A$	3.0	2.4	–	–
Parton shower on $\epsilon \times A$	7.8	29.6	–	–
*B*($H{\rightarrow }{\gamma \gamma }$)	2.1	2.1	2.1	2.1
*B*($H{\rightarrow }WW^*$)	1.5	1.5	1.5	1.5
Total	13.6	31.8	7.1	6.8

## Results

A fit to $m_{\gamma \gamma }$ is performed in the signal region to extract the signal yield. The statistical model is constructed with a likelihood function:

1 $$\begin{aligned} \mathcal {L}(\mu ,\varvec{\theta })&= \prod _{i} \Big ( ( n_{\mathrm {Signal}}(\mu ,\varvec{\theta }) + n_{\mathrm {ss}} ) \times f^{1}_{\mathrm {DSCB}}\left( m_{\gamma \gamma }^i,\varvec{\theta } \right) + n_{\mathrm {Cont}} \nonumber \\&\quad \times f_{\mathrm {Cont}}\left( m_{\gamma \gamma }^i,\varvec{\theta } \right) + n_{\text {SM-one-Higgs}}(\varvec{\theta }) \times f^{2}_{\mathrm {DSCB}}\left( m_{\gamma \gamma }^i,\varvec{\theta } \right) \nonumber \\&\quad + n_{\text {SM-di-Higgs}} \times f^{3}_{\mathrm {DSCB}}\left( m_{\gamma \gamma }^i,\varvec{\theta } \right) \Big ) \prod G(0| \varvec{\theta} 0, 1) \end{aligned}$$

- *i* stands for the event index,
- $n_{\mathrm {Signal}}$ is the expected number of signal events,
- $\mu$ is the cross section (times the branching fraction of $X\rightarrow HH$) of non-resonant (resonant) production,
- $n_{\mathrm {ss}}$ is the estimated spurious signal yield due to our choice of continuum background modelling,
- $f^{1}_{\mathrm {DSCB}}$ is the probability density function (pdf) of a double-sided Crystal Ball distribution for signal,
- $n_{\mathrm {Cont}}$ is the expected number of continuum background events,
- $f_{\mathrm {Cont}}$ is the pdf of the continuum background, i.e. an exponential function of a second-order polynomial,
- $n_{\text {SM-one-Higgs}}$ is the expected number of single-Higgs-boson events, which is set to the SM prediction and can vary with uncertainties,
- $f^{2}_{\mathrm {DSCB}}$ is the pdf of a double-sided Crystal Ball distribution for the SM single-Higgs-boson background,
- $n_{\text {SM-di-Higgs}}$ is the expected number of the SM di-Higgs-boson events,
- $f^{3}_{\mathrm {DSCB}}$ is the pdf of a double-sided Crystal Ball distribution for SM di-Higgs-boson background,
- $G(0|\varvec{\theta } 1)$ is the pdf of a Gaussian distribution used to constrain the nuisance parameters $\varvec{\theta }$ that model systematic uncertainties as introduced in Sect. 6.

Equation (1) is used directly for the BSM resonant signal searches. For the non-resonant SM Higgs boson pair search, the SM Higgs boson pair term is removed.

**Fig. 3:**
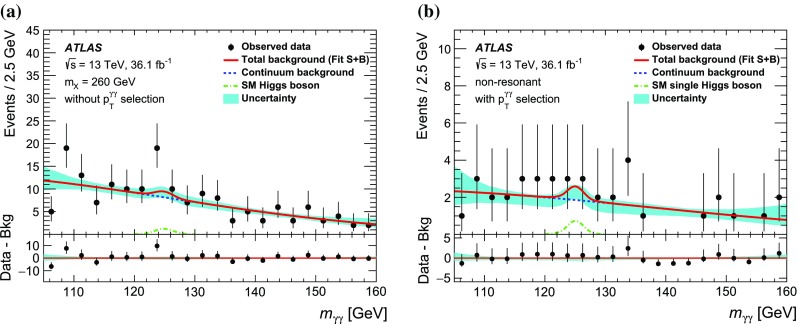
Invariant mass spectrum of the diphoton system in the searches for both resonant and non-resonant Higgs boson pair production, with the corresponding backgrounds for **a** $m_{X} = 260$ GeV without any $p_{\text {T}} ^{\gamma \gamma }$selection and **b** the non-resonant case with a $p_{\text {T}} ^{\gamma \gamma }$> 100 GeV selection. Fits to $m_{\gamma \gamma }$ are performed using the full signal-plus-background model. In each plot, only the background component is present. The shape parameters and normalisation of the continuum background model are determined in the fits. The “SM Higgs boson” in **a** contains the single-Higgs-boson background and SM di-Higgs-boson background. The band shows the uncertainty of the “Total background” in the upper panel and is calculated by a sampling method. The bottom panel shows the difference between the number of events in data and the estimated number of background events, as determined by the fits

**Fig. 4:**
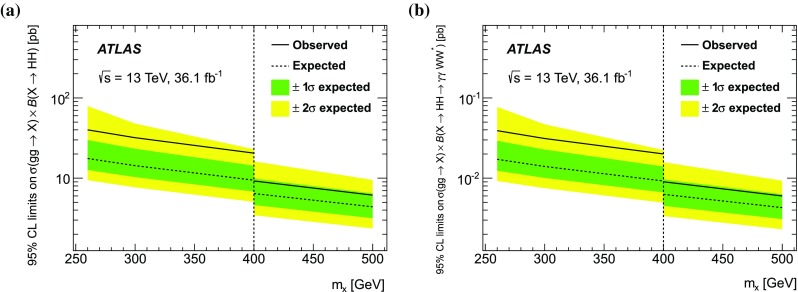
$95\%$ CL expected (dashed line) and observed (solid line) limits on the resonant Higgs boson pair production cross section times the branching fraction of $X{\rightarrow }HH$ as a function of $m_X$ (**a**) with and **b** without assuming the SM branching fractions of $H{\rightarrow }WW^*$ and $H{\rightarrow }{\gamma \gamma }$. The $\text {CL}_\text {s}$ method and the asymptotic approximation are used. The $\pm 1\sigma$ and $\pm 2\sigma$ bands on the expected limit are also presented. To the right of the vertical dashed line at $m_{X} = 400$ GeV, the $p_{\text {T}} ^{\gamma \gamma } > 100$ GeV selection is applied in both plots, but not to the left

The distributions in the final signal-plus-background fit using the likelihood function above are shown for two sets of selections separately: in Fig. 3a without requiring the $p_{\text {T}} ^{\gamma \gamma }$selection for masses below 400 GeV, and in Fig. 3b requiring $p_{\text {T}} ^{\gamma \gamma }$> 100 GeV for masses above 400 GeV, as well as for the search for non-resonant Higgs boson pair production. The fits are performed separately on the two distributions to search for resonant signals in both the low-mass and high-mass ranges. The observed data are found to be compatible with the sum of the expected SM backgrounds by performing a likelihood-ratio test [92]. The largest data excess has a local significance of 2.0 standard deviations at 400 GeV without the $p_{\text {T}} ^{\gamma \gamma }$selection. A modified frequentist method $\text {CL}_\text {s}$ [93] is used to calculate the 95% confidence-level (CL) exclusion limits with the asymptotic approximation [92]. Unfolding the SM Higgs boson branching fractions to $WW^*$ and $\gamma \gamma$ for the signal, the expected upper limit on the cross section for non-resonant Higgs boson pair production is 5.4 pb, while the observed limit is 7.7 pb, as shown in Table 6. The difference between the expected and observed limits is due to a slight excess of events in data. The expected upper limit on the cross section times the branching fraction of $X\rightarrow HH$ ranges from 17.6 to 4.4 pb, while the observed limit ranges from 40 to 6.1 pb, as a function of $m_X$ between 260 and 500 GeV, as shown in Fig. 4a.

**Table 6:** The 95% CL upper limits for the non-resonant production and the ratios of the limits to the SM cross-section value of $\sigma (pp\rightarrow HH)=33.4^{+2.4}_{-2.8}$ fb [17]. The $\pm 1\sigma$ and $\pm 2\sigma$ intervals around the median limit are also presented

	$+2\sigma$	$+1\sigma$	Median	$-1\sigma$	$-2\sigma$	Observed
Upper limits on $\sigma (HH)$ (pb)	12	8.0	5.4	3.9	2.9	7.7
Upper limits on $\sigma (HH)\times B(\gamma \gamma WW^{*})$ (fb)	12	7.8	5.3	3.8	2.8	7.5
Ratios of limits over the SM $\sigma (HH)$	360	240	160	120	87	230

Assuming the SM Higgs branching fractions of $B(H{\rightarrow }WW^*)=(21.52\pm 0.32)\%$ and $B(H{\rightarrow }{\gamma \gamma })=(0.227\pm 0.005)\%$ [17], the expected upper limit on the cross section for non-resonant production of $HH\rightarrow \gamma \gamma WW^*$ is 5.3 fb, while the observed limit is 7.5 fb, as shown in Table 6. The expected upper limit on the cross section for resonant production of $X\rightarrow HH\rightarrow \gamma \gamma WW^*$ ranges from 17.2 to 4.3 fb, while the observed limit ranges from 39.1 to 6.0 fb, as a function of $m_X$ between 260 and 500 GeV, as shown in Fig. 4b. The statistical uncertainty dominates in the final limits, while the impact of systematic uncertainties on these limits is only a few percent.

## Conclusion

This paper presents searches for non-resonant and resonant Higgs boson pair production with a semileptonic $\gamma \gamma WW^*$ final state using 36.1 $\hbox {fb}^{-1}$ of $pp$ collision data collected at 13 TeV with the ATLAS detector at the LHC. No significant excess above the expected SM background is observed. A 95% confidence-level upper limit of 7.7 pb is set on the cross section for non-resonant production, while the expected limit is 5.4 pb, compared to the SM Higgs boson pair production cross section of 33.4 fb. The observed upper limit on the resonant production cross section times the branching fraction of $X\rightarrow HH$ ranges between 40 pb and 6.1 pb, while the expected limit ranges between 17.6 and 4.4 pb, for a hypothetical resonance with a mass in the range of 260–500 GeV. When deriving the limits above, the SM branching ratios of the $H{\rightarrow }{\gamma \gamma }$ and $H{\rightarrow }WW^*$ are assumed.

## Footnotes

1 ATLAS uses a right-handed coordinate system with its origin at the nominal interaction point (IP) in the centre of the detector and the *z* axis along the beam pipe. The *x* axis points from the IP to the centre of the LHC ring, and the *y* axis points upwards. Cylindrical coordinates $(r,\phi )$ are used in the transverse plane, $\phi$ being the azimuthal angle around the *z*-axis. The pseudorapidity is defined in terms of the polar angle $\theta$ as $\eta = -\ln \tan (\theta /2)$. Angular distance is measured in units of $\Delta R \equiv \sqrt{{(\Delta \eta )}^{2} + {(\Delta \phi )}^{2}}$.

2 The tune parameters can be found at the following link: https://herwig.hepforge.org/tutorials/mpi/tunes.html

3 Converted photons are those that convert to an $e^{+}e^{-}$ pair inside the ID volume, with at least one of the two lepton trajectories reconstructed, while unconverted photons directly enter the EM calorimeter.
